# Improvement of Game Users’ Depressive Symptoms via Behavioral Activation in a Massive Multiplayer Online Game: Randomized Controlled Trial

**DOI:** 10.2196/73734

**Published:** 2025-09-24

**Authors:** Kenji Yokotani, Masanori Takano, Nobuhito Abe, Takahiro A Kato

**Affiliations:** 1 Graduate School of Technology, Industrial and Social Sciences, Tokushima University, Minamijosanjimacho 1-1, Tokushima, 770-8502, Japan, 81 88-656-7000; 2 Department of Data Science, PsychoBit, Inc., Kobe, Japan; 3 Multidisciplinary Information Science Center, CyberAgent (Japan), Tokyo, Japan; 4 Institute for the Future of Human Society, Kyoto University, Kyoto, Japan; 5 Department of Psychiatry, Hokkaido University Graduate School of Medicine, Sapporo, Japan

**Keywords:** massive multiplayer online games, behavioral activation, exposure therapy, depression, social anxiety

## Abstract

**Background:**

Online games developed to improve mental health symptoms are reportedly effective among game users. However, it has not been verified whether massive multiplayer online games (MMOGs) developed for leisure purposes are effective in improving users’ mental health symptoms.

**Objective:**

Based on 2 theoretical frameworks, this study examined whether MMOGs improve depression and social anxiety. First, behavioral activation theory posits that depressive symptoms improve through the repetition of reward-linked behaviors. Second, inhibitory learning theory suggests that exposure to social stimuli (eg, being the center of attention) previously perceived as threatening reduces fear responses over time.

**Methods:**

Participants were Pigg Party users with at least 3 months of previous experience. Overall, 1105 participants were randomly assigned to either the experimental (n=548) or waitlist groups (n=557). Participants in the experimental group were instructed to ring a friend’s room bell (an action that clearly draws attention) on weekdays and to customize their avatars on weekends. Those completing ≥60% of the tasks received additional monthly rewards. The waitlist group received no interventions but was given random additional rewards. Both groups completed questionnaires on depression (Quick Inventory of Depressive Symptomatology) and social anxiety (Brief Liebowitz Social Anxiety Scale) at baseline, and again at 1, 2, and 3 months.

**Results:**

The experimental group showed a significantly higher frequency of bell ringing compared to that of the waitlist group (standardized mean difference [SMD]=0.13), whereas no significant difference was observed between the groups in avatar customization frequency. As predicted, the experimental group showed a significantly greater reduction in depressive symptoms, with a small effect size observed (SMD=–0.12). However, no significant difference was determined between groups in social anxiety symptoms.

**Conclusions:**

This study demonstrated that MMOGs, when combined with administrator-led interventions, can reduce users’ depressive symptoms, albeit with a small effect size. Further studies are needed to test the intervention effects on social anxiety symptoms in MMOGs, with improved exposure scenarios.

## Introduction

Interventions using online games to improve the mental health symptoms of game users have been increasingly implemented in recent years [1]. These online games reportedly improve depression [2] and anxiety [3] symptoms. Although most of these online games have been developed to improve mental health symptoms, massive multiplayer online games (MMOGs) developed for leisure purposes [4] might have similar effects on depressive [5] and anxiety symptoms [6]. In fact, user networks within MMOGs represent sources of social support [7], and gameplay experiences in MMOGs are associated with subjective well-being [8].

**Figure 1.**
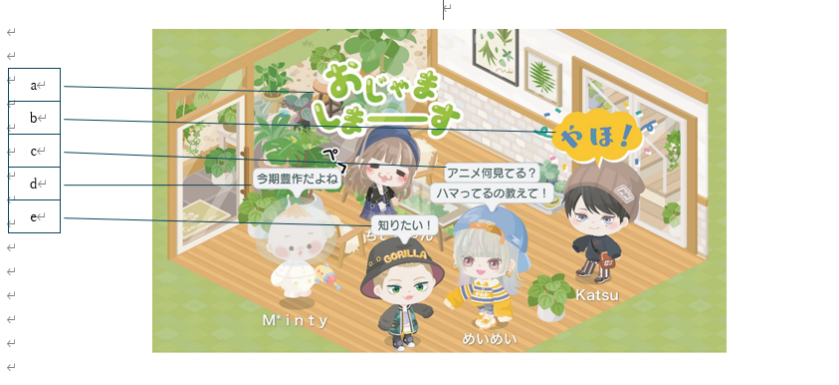
Players’ behavior in the Pigg Party. Each player has an avatar and a private room, both of which can be customized to visit the private rooms of other players. Permission must be obtained from the owner in advance to visit a private room. Translation of the figure. (a) “I’m going to enter the room.” (b) “Hi.” (c) “What anime do you watch? Tell me what you are into.” (d) “It’s a bumper crop this season.” (e) “I want to know that too.”

We especially focused on the Pigg Party (Figure 1), a well-known MMOG in Japan, which has been linked to extensive mental health research. For instance, previous studies have shown that bipolar disorder [9], depression [10], and social anxiety symptoms [11] can be estimated from user interactions in Pigg Party. These studies suggested that interaction logs in the Pigg Party contained indicators of mood and anxiety disorders. Moreover, online support within the Pigg Party had been reported to represent users’ social resources [12], and these resources were suggested to reduce users’ risk of mental illness [6]. Briefly, while user interactions in the Pigg Party may serve as indicators of mental illness, the support provided within the Pigg Party has the potential to mitigate those risks. Therefore, one randomized control trial in Pigg Party suggested that user interactions in the game may alleviate users’ depressive symptoms [5]. However, the sample size for this trial was small, and the completion rate of the experimental task was not high. Therefore, this study will examine whether completing tasks that are easy to perform within the Pigg Party, with a sample size of over 1000 participants, contributes to the improvement of users’ depressive and anxiety symptoms.

This study used 2 theoretical frameworks. First is the behavioral activation of depressive symptoms [13]. Individuals with depressive symptoms have difficulty learning the association between their behaviors and the rewards linked to them [14], which makes it challenging for them to experience positive emotional states and facilitates the maintenance of these symptoms [15]. By increasing behaviors associated with rewards, they can learn reward-associated behaviors, experience positive emotional states, and improve depressive symptoms [16]. Meta-analysis of behavioral activation shows that activation of reward-associated behaviors improves depressive symptoms [17]. Activation of reward-associated behaviors—implemented through online games, such as webpage applications [18], short message services [19], and smartphone apps [20]—has demonstrated efficacy in improving depressive symptoms. Activation of reward-associated behaviors within online games also improved users’ depressive symptoms [21]. Indeed, reciprocal reward relationships—such as mutually exchanging “likes” in an MMOG—can reduce depressive symptoms [5]. These studies suggest that the activation of reward-associated behaviors in MMOGs improves the depressive symptoms of game users [5].

Online games also improve the anxiety symptoms of game users [3].

Inhibitory learning theory for anxiety symptoms serves as our second theoretical framework [22]. According to this theory, repeated relearning—where individuals who perceive specific stimuli (eg, being the focus of attention) as threatening reinterpret them as safe—reduces the perceived danger of those stimuli [23]. This theory is empirically supported by exposure therapy. Individuals with social anxiety symptoms tend to become anxious because they have learned to perceive social situations as dangerous stimuli [24]. By repeatedly experiencing social situations with little danger, they learn to perceive such situations as safe stimuli, which in turn improves their anxiety symptoms [25]. Their relearning of social situations has been confirmed in neuropsychological experiments [26], and the effectiveness of exposure therapy in improving social anxiety symptoms has been widely demonstrated [27]. Virtual reality exposure therapy, which constructs a virtual social community within online games and enables users to repeatedly experience social situations in the virtual community, has shown high intervention efficacy for social anxiety [28]. Virtual reality exposure therapy has been demonstrated to be effective for social anxiety symptoms in children [29], adolescents [30], and adults [31]; therefore, various virtual communities have been developed [32]. These findings suggest that repetitive experience of virtual social situations improves the social anxiety of game users [6].

**Figure 2.**
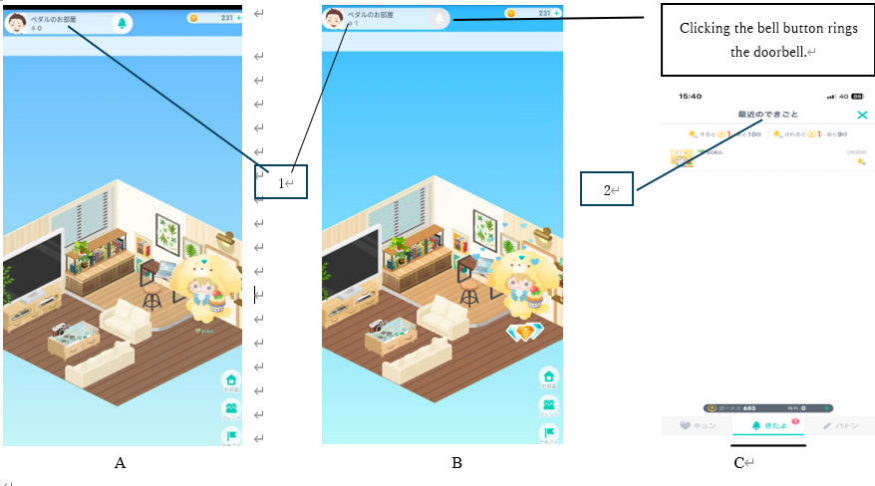
Process of entering another user’s room and ringing the doorbell on the Pigg Party. (A) One user visits another user’s room, (B) One user rings the doorbell of another user’s room. (C) The owner of the room can see who rang the doorbell. 1: “Pedal Room” (where “Pedal” refers to a person’s name); 2: “Recent Events.”

In exposure therapy, it is essential to provide exposure scenarios in which participants experience fear [33]. Individuals with social anxiety disorder fear situations in which they are the focus of attention by others [34]; therefore, it is necessary to provide such attention-focused situations as the exposure scenarios. In a Pigg Party task where the player rings a bell in a friend’s room, the player must first enter the room before ringing the bell. Upon entry, they become the focus of attention from people already in the room (Figure 1). Furthermore, when the bell is rung, the ringer is highlighted with an animation, drawing even more attention (Figure 2B). Thus, the bell-ringing task includes situations where the individual is noticed both upon entry and at ringing the bell, making it an appropriate exposure situation for individuals with social anxiety disorder. In fact, individuals with social anxiety disorder avoid attention even in the MMOG [11], and the situation where they receive attention from others is set as the exposure scenario [35].

Based on these studies, we created an experimental group in which game users repetitively experienced social situations in an MMOG. Specifically, in Pigg Party, users in the experimental group were prompted to engage in the behavior of ringing a doorbell in another user’s private room as the exposure scenarios (Figure 2). In addition, users were prompted to regularly customize their own avatars (Multimedia Appendix 1), enhancing self-identification with their avatars [6,36] and making social behavior in MMOG more analogous to social behavior in a physical community [37]. These prompts enabled the experimental group to receive virtual reality exposure therapy in the Pigg Party. Furthermore, by providing monetary rewards for ringing the doorbell, the behavior of ringing the doorbell becomes a reward-associated behavior in an MMOG [38]. In contrast, the waitlist group did not receive any intervention but did receive a random reward. As a result, the experimental group learned reward-associated behaviors, but the waitlist group did not. Thereby, only the experimental group received behavioral activation for reward-associated behaviors in the MMOG.

Our hypotheses are as follows: (1) according to behavioral activation on depressive symptoms [13,21], the experimental group would exhibit improvements in depressive symptoms compared to the waitlist group. (2) According to inhibitory learning theory on social anxiety symptoms [28,39], the experimental group would exhibit improvements in social anxiety, both within an MMOG and in the physical community, compared to that of the waitlist group. In addition, we examined whether Hypotheses 1 and 2 would be supported among individuals with depressive disorder and those with social anxiety disorder.

## Methods

### Design

This study involved a single-site, randomized, parallel-group comparative trial (Trial Registration: wdb9u). The study protocol was approved by the ethics committee of the National University A in Japan. The detailed protocol for this experiment has been disclosed previously [40].

### Participants

The participants of this study were users who had been using the Pigg Party before November 15, 2023. Pigg Party is a mobile game that can be played on Android and iPhone devices, but its development environment has not been disclosed. On December 5, 2023, the administrator of the Pigg Party emailed the users requesting their participation in the study. By December 15, 2023, 1331 users had expressed their willingness to participate, and then their eligibility for participation was assessed. As a result, 226 users were removed from the participants; among these, 175 users had withdrawn their consent to participate in the experiment, and after the recruitment period, 51 users requested to participate. Eligibility criteria required users to have played Pigg Party by November 15, 2023, and to understand Japanese. All users who completed the first questionnaire met both criteria.

The final sample consisted of 1105 Pigg Party users. Participants’ mean age was 23.68 (SD 10.44) years, ranging from 9 to 82 years. No exclusions were made based on participants’ age or severity of psychiatric symptoms. Baseline social anxiety symptoms were assessed using the Japanese version of the Brief Liebowitz Social Anxiety Scale (Brief LSAS-J) [6,41]. Because the Brief LSAS-J includes 14 items, whereas the original LSAS contains 24, raw scores were multiplied by 24/14 to ensure comparability with the full LSAS metric [6,41]. Applying the established clinical cut-off of 30 points [42,43] to the adjusted scores indicated that 944 individuals (944/1105, 85.4 %) met the threshold for social anxiety; thus, the majority of the sample can be classified within the social anxiety disorder spectrum (Multimedia Appendix 2). To assess the depressive symptoms of participants, the Japanese version of the Quick Inventory of Depressive Symptomatology (QIDS-J) was used [44,45]. The QIDS-J is a 16-item questionnaire that uses a 4-point Likert scale, with higher scores indicating greater severity of depression. Applying the established clinical cut-off of 6 points [46,47] revealed that 677 individuals (677/1105, 61.2%) met the criterion for depression. Consequently, more than half of the present sample can be classified within the depressive disorder spectrum (Multimedia Appendix 3).

### Randomization and Masking

**Figure 3.**
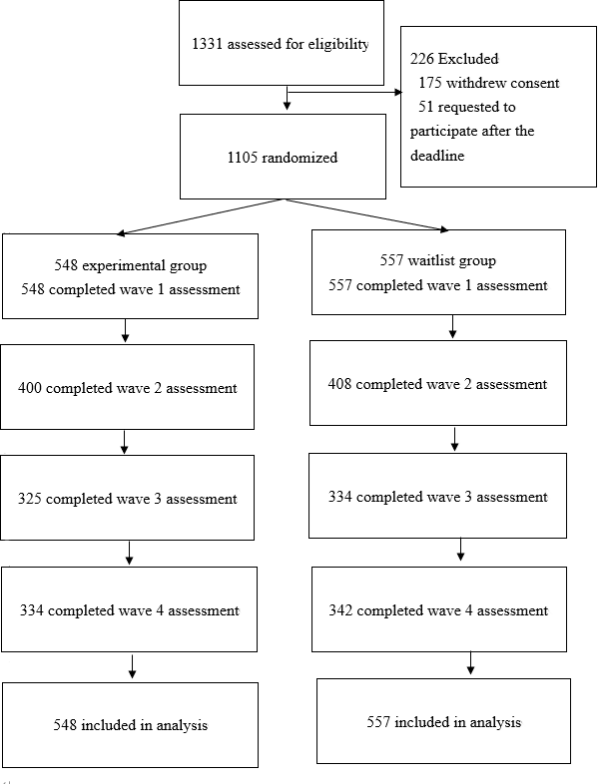
Participant flow diagram.

A total of 1105 participants were randomly assigned to the experimental and waitlist groups in a 1:1 ratio (Figure 3). The Mersenne Twister method [44], which is implemented by default in R, was used for the random assignment. As the participants’ Pigg Party tasks differed depending on the assigned group, assigned group masking was not implemented for the participants.

### Procedures

Participants completed questionnaires on demographic characteristics and outcome measures of mental health symptoms in the first wave (baseline, December 5‐15, 2023). In the second wave (January 16‐26, 2024), third wave (February 16‐26, 2025), and fourth wave (March 16‐26, 2025), they completed questionnaires solely on outcome measures of mental health symptoms. Participants who completed the questionnaires for the first and fourth waves within the designated period were provided with an Amazon gift voucher worth JPY 500 (US $3.39) or a Google Play gift voucher, whereas those who completed the questionnaires for the second and third waves within the designated period were provided with an Amazon gift voucher worth JPY 200 (US $1.36) or a Google Play gift voucher. Each questionnaire was available for 10 days, and the intervention lasted 30 days, with these periods alternating (Multimedia Appendix 4).

**Figure 4.**
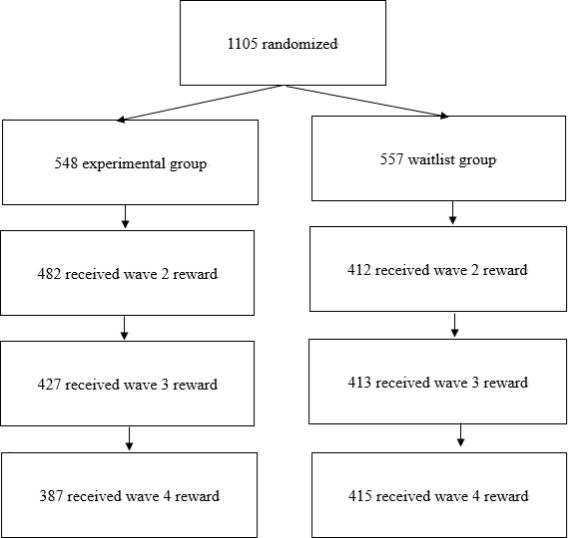
The number of participants who received additional rewards in the experimental and waitlist groups. The experimental group received additional rewards for completing 60% or more of the tasks, whereas the waitlist group received additional rewards through a lottery.

The experimental group was prompted to visit another user’s room once per day and ring the bell on weekdays (Monday through Friday, excluding public holidays; refer to Figure 2 for the method of ringing the bell) during the 3-month experimental period (from December 16, 2023, to March 15, 2024). In addition, on weekends (Saturday and Sunday) or public holidays, they were prompted to customize their avatar once per day (Multimedia Appendix 1 for the method of customization). Participants who accomplished these tasks on at least 18 days (18/30, 60%) out of 30 days were awarded an additional bonus in the form of an Amazon or Google Play gift voucher worth JPY 500 (US $3.39). As the experimental period spanned three months, gift vouchers worth a maximum of JPY 1500 (US $10.24) were provided as additional bonuses per user. Figure 4 shows the number of participants in the experimental group who received additional rewards.

In contrast, the waitlist group received no prompts during the experimental period. Within the waitlist group, 73% (412-415/557) of the users were randomly selected each month and were awarded an additional bonus of either Amazon or Google Play gift vouchers worth JPY 500 (US $3.39; Figure 4). One previous study on Pigg Party [5] reported that 73% of participants in the experimental group continued the intervention (sending “likes” to other users on at least 18 of 30 d). Therefore, we assumed a similar proportion would receive rewards in the experimental group in the present study and offered the same proportion to the waitlist group. Since the experimental period lasted 3 months, gift vouchers worth a maximum of JPY 1500 (US $10.24) were provided as additional bonuses per user. Figure 4 illustrates the number of participants in the waitlist group who received additional rewards.

### Outcome Measures

To assess depressive symptoms, the QIDS-J was used [45,46]. The QIDS-J is a 16-item questionnaire that uses a 4-point Likert scale, with higher scores indicating greater severity of depression. The Cronbach α coefficient for the QIDS-J at baseline was 0.86.

To assess social anxiety symptoms, the Brief LSAS-J was used [6,41]. The Brief LSAS-J can measure social anxiety symptoms in both virtual and physical communities. The term “virtual community” refers to the Pigg Party, whereas “physical community” denotes face-to-face interactions. Each version of the questionnaire comprises 14 items that assess anxiety symptoms and avoidance behaviors within the respective community. Details of the questionnaire are provided in Multimedia Appendix 5. Higher scores on the Brief LSAS-J indicate greater severity of social anxiety symptoms in both virtual and physical communities. The Cronbach α coefficients for the Brief LSAS-J at baseline in virtual and physical communities were 0.95 and 0.97, respectively.

### Game Behavioral Logs for Manipulation Checks

To verify the frequency with which users rang the bell, it was measured monthly. Since the frequency of bell ringing is proportional to the number of friends and follows a power-law distribution (Multimedia Appendix 6), the value was calculated by adding one and then applying a base 2 logarithmic transformation.

Similarly, to verify the frequency with which users changed their avatars’ clothing, it was measured monthly. As with the frequency of bell ringing, avatar customization frequency followed a power-law distribution (Multimedia Appendix 7). The resulting frequencies were transformed by adding one and applying a base 2 logarithmic transformation.

### Statistical Analysis

The final number of participants required was 1043 (based on an estimate for the latent growth model with Cohen *d*=0.10, *α*=.05, *β*=.90) [47,48]. In latent growth model sample size estimation, priority is given to the number of participants in each group at the final time point, rather than to the total number of measurement points; thus, the number of time points does not influence the required sample size or statistical power [49]. After recruiting 1105 participants, it was deemed that a sufficient sample size had been reached, and further recruitment was terminated.

Latent growth curve modeling (LGCM) was used in this study. The current structural equations of the LGCM accounted for the effect of gender, following a previous study [50]. The equations are as follows:


(1) Y[it]=B0[i]+B1[i]F[t]+E2[it]

(2) B0[i]=B00+B0exp∗Gexp[i]+B0female∗Gfemale[i]+E0[i]

(3) B1[i]=B10+B1exp∗Gexp[i]+B1female∗Gfemale[i]+E1[i]

Where Y[it] represents the outcome measures (depressive symptoms, social anxiety symptoms) at time t for participant i. B0[i] and B1[i] are the intercept and slope contributing to Y[it] for participant i. E2[it] is the error contributing to Y[it] at time t for participant i . In line with previous studies [51], F[t] is obtained by logarithmically transforming the time of 4t weeks after adding 1. Specifically, for 4*t*=0, 4, 8, and 12 weeks, F[t] corresponds to 0.000, 1.000, 1.585, and 2.000, respectively. Improvement in psychiatric symptoms tends to follow a pattern in which there is a rapid improvement at the initial stage, followed by a gradually diminishing but sustained effect over time [52]. This pattern approximates the graph of a logarithmic function with a base>1 (ie, large initial change followed by a decelerated rate of change). Accordingly, many LGCMs for psychiatric symptoms use log-transformed time variables [53], and this study follows that convention.

The intercept B0[i] is influenced by B00, B0exp, B0female, and E0, which represent the intercept for the intercept, the intercept effect of the experimental group, the intercept effect of the female group, and the intercept effects of individuals, respectively. Gexp[i] is a group variable indicating whether participant i belongs to the experimental group (1 if yes, 0 if no). Similarly, Gfemale[i] is another group variable indicating the sex of the participant (1 for females, 0 for no females).

The slope B1[i] is influenced by B10, B1exp, B1female, and E1, which represent the intercept for the slope, slope effect of the experimental group, slope effect of the female group, and slope effect of individuals, respectively. B1exp represents the time-variant effects of the experimental group on the outcome measure Y[it]. Hence, this effect represents the intervention effects of the experimental group on the outcome measures. The intervention effects were analyzed using 95% CI and Wald tests. The Widely Applicable Bayesian Information Criterion (WAIC) and Leave-One-Out cross-validation (LOO) were used as indicators of the model’s overall fit.

To estimate the intervention’s effect size, the standardized mean difference (SMD) was calculated using the following formula, which is identical to Cohen *d* and widely used [54].


(4) $$\frac{\left({Wave4}_{exp}-{Wave1}_{exp}\right)-\left({Wave4}_{wait}-{Wave1}_{wait}\right)}{\sqrt{\frac{\left(n_{exp}-1\right)\sigma_{exp}^{2}+\left(n_{wait}-1\right)\sigma_{wait}^{2}}{n_{exp}+n_{wat}-2}}}$$

Where Wave4exp and Wave1exp represent average outcome measure at waves 4 and 1 in the experimental group. Wave4wait and Wave1wait represent average outcome measure at waves 4 and 1 in the waitlist group. The nexp and nwait represent the number of participants in the experiment and waitlist groups, respectively. The σexp is the standard deviation of the difference between outcome measure waves 4 and 1 in the experimental group. Similarly, σwait is the SD of the difference between outcome measure waves 4 and 1 in the waitlist group.

In accordance with clinical trial guidelines [55], intention-to-treat analyses were performed. Full Information Maximum Likelihood (FIML) was used to handle missing data [56], as it is commonly used for estimating missing data in clinical trials [50]. During the FIML procedure, 0.0001 was added to the diagonal matrix to ensure that the covariance matrix remained positive. In addition, instead of an inverse matrix, a pseudo-inverse matrix was used to manage errors when the inverse matrix did not exist. The initial values were set as the mean of each variable, and the optimization process was accelerated using the Limited-memory Broyden-Fletcher-Goldfarb-Shanno algorithm-bound optimization approach [57].

An independent-samples *t* test was conducted to compare the experimental and waitlist groups at baseline, with Cohen *d* reported as the effect size. Pearson’s correlation coefficient was used for correlation analyses. A *P* value of <.05 was considered statistically significant.

### Ethical Considerations

This study was approved by the ethics committee of the National University A in Japan as of December 1, 2023 (registration number 301). Informed consent was obtained from all the participants in this study. All procedures were conducted in accordance with the revised 1964 Helsinki Declaration and its later amendments, or comparable ethical standards. The participants’ data were anonymized. Participants received either an Amazon or Google Play gift card as compensation for completing the questionnaires. The amounts were as follows in JPY (where JPY 150 is equivalent to around US $1): JPY 500 (US $3.39) for the first round, JPY 200 (US $1.36) for the second, JPY 200 (US $1.36) for the third, and JPY 500 (US $3.39) for the fourth. In addition, during the intervention phase, participants received a JPY 500 (US $3.39) bonus for each of the first 3 rounds. Thus, participants received a minimum of JPY 500 (US $3.39) and a maximum of JPY 2900 (US $19.80) for their participation in the experiment. No personally identifiable information appears in the data, figures, or tables of this study.

## Results

### Comparison of Demographic and Outcome Variables at Baseline Between the Experimental and Waitlist Groups

Before hypothesis testing, the demographic and outcome variables at the baseline of the experimental and waitlist groups were compared. In the experimental group, the proportion of females was significantly lower than that in the waitlist group (Table 1). The phi coefficient was 0.06, indicating a smaller-than-small effect (small=0.10); however, no significant differences were observed in other demographic variables, such as age, educational status, and employment status (Table 1). Further, no significant differences were found when comparing depressive, virtual social anxiety, and real-life social anxiety symptoms at baseline survey (Table 1). These results indicate that, aside from the proportion of females, the experimental and waitlist groups were evenly distributed.

**Table 1.** Comparison of demographic and outcome variables at baseline between the experimental and waitlist groups. .

	Experimental group (n=548)	Waitlist group (n=557)	Statistics				
			*t* test (*df*)	Chi-square (*df*)	*P* value	Cohen d	φ
Age (years), mean (SD)	24.151 (10.528)	23.226 (10.347)	–1.473 (1101.7)	—^a^	.141	0.089	—
Female, n (%)	394 (0.719)	432 (0.776)	—	4.394 (1)	.036^b^	—	0.063
Retired, n (%)	1 (0.002)	0 (0)	—	0 (1)	.993	—	0.000
Homemaker, n (%)	53 (0.097)	49 (0.088)	—	0.159 (1)	.691	—	0.012
Employed, n (%)	203 (0.37)	186 (0.334)	—	1.458 (1)	.227	—	0.036
On leave from studies or work, n (%)	22 (0.04)	26 (0.047)	—	0.148 (1)	.700	—	0.012
Student, n (%)	244 (0.445)	277 (0.497)	—	2.798 (1)	.094	—	0.050
Unpaid family worker, n (%)	29 (0.053)	24 (0.043)	—	0.389 (1)	.533	—	0.019
Preparing for university entrance or employment, n (%)	8 (0.015)	13 (0.023)	—	0.712 (1)	.399	—	0.025
Unemployed, n (%)	46 (0.084)	46 (0.083)	—	0 (1)	>.99	—	0.000
On long-term vacation, n (%)	15 (0.027)	17 (0.031)	—	0.018 (1)	.894	—	0.004
Not falling under any of the above categories, n (%)	12 (0.022)	22 (0.039)	—	2.309 (1)	.129	—	0.046
Outcome variables at baseline							
Depressive symptoms, mean (SD)	8.78 (6.06)	8.1 (5.99)	–1.903 (1103)	—	.057	0.114	—
Social anxiety symptoms in virtual, mean (SD)	28.95 (18.97)	28.39 (19.07)	–0.486 (1103)	—	.627	0.029	—
Social anxiety symptoms in physical, mean (SD)	46.81 (23.49)	45.66 (23.22)	–0.817 (1103)	—	.414	0.049	—

a Not applicable.

b *P*<.05.

### Verification of the Experimental Manipulation

**Figure 5.**
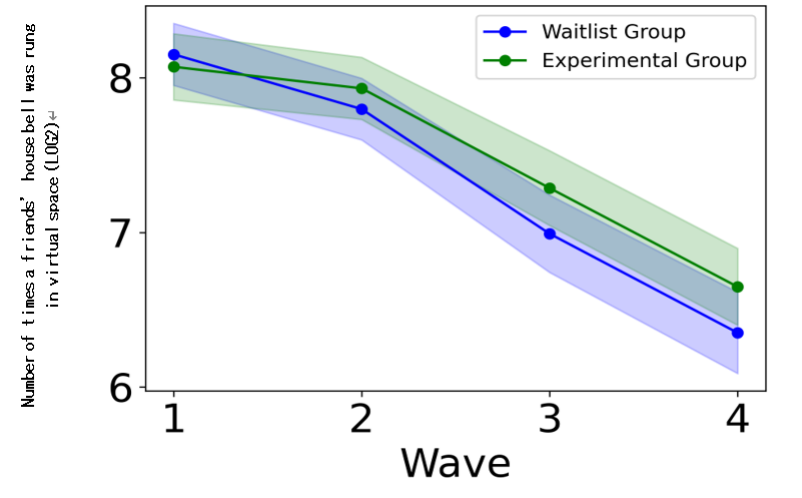
Comparison of the number of times a friend’s house bell was rung in a virtual space between the experimental and waitlist groups (LOG2). The shaded area around the line represents the 95% confidence interval. As the y-axis values of 8, 7, and 6 are plotted on a base-2 logarithmic scale, they correspond to actual bell ring counts of 256, 128, and 64, respectively.

In addition, we confirmed that the manipulation applied to the experimental group was conducted appropriately. The experimental group exhibited a significantly higher number of bell-ringing behaviors than the waitlist group (Table 2). The effect size was 0.13, confirming a small effect (Table 2). Figure 5 also shows that although there was almost no difference in the number of bell-ringing behaviors during the first wave (baseline), the experimental group exhibited a higher number of bell-ringing behaviors during the second, third, and fourth waves. In contrast, no significant difference was observed in the frequency of avatar customization between the experimental and waitlist groups (Table 2). Because the prompt for ringing the bell occurred on weekdays (5/7 d) and the prompt for customization occurred on weekends (2/7 d), the frequency of prompts may have influenced the experimental manipulation. These results indicate that the prompt for ringing the bell on the MMOG in the experimental group was sufficiently implemented, whereas the prompt for customizing the avatar was not.

**Table 2.** Intention-to-treat analysis of latent growth curve modeling of online game behaviors in the overall sample. The Gelman-Rubin statistic (rhat) for all coefficients was 1.0.

		Intervention effect					Baseline (Wave1)		Wave 2		Wave 3		Wave 4		SMD^a^
		β	95% LL^b^	95% UL^c^	Wald	*P* value	mean (SD)		mean (SD)		mean (SD)		mean (SD)		
The number of bells rang (LOG2)^d^															
	Experiment	0.195	0.031	0.36	2.321	0.020	8.07 (2.56)		7.93 (2.4)		7.29 (2.89)		6.65 (2.97)		0.13
	Waitlist	—^e^	—	—	—	—	8.15 (2.42)		7.8 (2.4)		6.99 (3.01)		6.35 (3.17)		—
The number of avatar customizations (LOG2)^f^															
	Experiment	0.069	-0.065	0.197	1.03	0.303	7.71 (1.67)		7.56 (1.92)		6.5 (2.12)		6.13 (2.29)		0.07
	Waitlist	—	—	—	—	—	7.65 (1.7)		7.48 (1.9)		6.39 (2.05)		5.9 (2.37)		—

a SMD: standardized mean difference.

b LL: lower limit.

c UL: upper limit.

d Widely Applicable Bayesian Information Criterion (WAIC) -8376.6 leave-one-out (LOO) -8544.8.

e Not applicable.

f WAIC -7392.2, LOO -7523.4.

### Examination of the Intervention Effects on Depressive Symptoms

**Figure 6.**
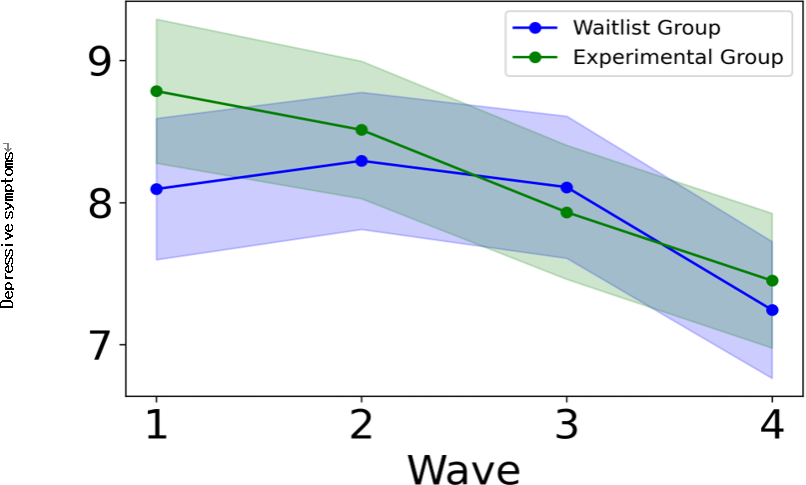
Comparison of depressive symptoms between experimental and waitlist groups. The shaded area around the line represents the 95% CI.

We compared depressive symptoms between the experimental and waitlist groups. The experimental group exhibited a significantly greater improvement in depressive symptoms compared to the waitlist group (Table 3). The effect size was −0.12, indicating a small effect (Table 3). Figure 6 further illustrates that the experimental group demonstrated a consistent improvement in depressive symptoms. These findings support hypothesis 1.

**Table 3.** Intention-to-treat analysis of latent growth curve modeling of depressive symptoms in the overall sample^a^. The Gelman–Rubin statistic (rhat) for all coefficients was 1.0.

Depressive symptoms	Intervention effect					Baseline (Wave 1), mean (SD)	Wave 2, mean (SD)	Wave 3, mean (SD)	Wave 4, mean (SD)	SMD^b^
	B	95% LL^c^	95% UL^d^	Wald	*P* value					
Experiment	–0.319	–0.543	–0.08	–2.703	.007^e^	8.78 (6.06)	8.51 (5.78)	7.93 (5.63)	7.45 (5.66)	–0.12
Waitlist	—^f^	—	—	—	—	8.1 (5.99)	8.29 (5.81)	8.11 (6.02)	7.24 (5.8)	—

a Widely Applicable Bayesian Information Criterion (WAIC) -10658.7, leave-one-out (LOO) -10801.2.

b SMD: Standardized Mean Difference.

c LL: lower limit.

d UL: upper limit.

e *P*<.01.

f Not applicable.

In addition, we tested hypothesis 1 among participants with depressive disorder only (n=677; QIDS-J ≥6). As presented in Table 4, when restricting the sample to individuals with depressive disorder, no significant difference in depressive symptoms was observed between the experimental and waitlist control groups. This finding suggests that, although behavioral activation delivered via an online game may reduce depressive symptoms in the overall sample, its disorder-specific effectiveness among individuals with depressive disorder may be limited.

**Table 4.** Intention-to-treat analysis of latent growth curve modeling of depressive symptoms in individuals with depressive disorder^a^. The Gelman–Rubin statistic (rhat) for all coefficients was 1.0. The number of participants in the experiment and waitlist groups was 346 and 331, respectively.

Depressive symptoms^a^	Intervention effect					Baseline (Wave 1), mean (SD)	Wave 2, mean (SD)	Wave 3, mean (SD)	Wave 4, mean (SD)	SMD^b^
	B	95% LL^c^	95% UL^d^	Wald	*P* value					
Experiment	–0.28	–0.61	0.04	–1.64	.10	12.27 (4.86)	11.31 (5.29)	10.53 (5.25)	9.86 (5.53)	–0.08
Waitlist	—^e^	—	—	—	—	11.90 (4.75)	11.09 (5.37)	10.96 (5.74)	9.83 (5.75)	—

a Widely Applicable Bayesian Information Criterion (WAIC) -6716.6, leave-one-out (LOO) -6799.8.

b SMD: standardized mean difference.

c LL: lower limit.

d UL: upper limit.

e Not applicable.

### Examination of the Intervention Effects on Social Anxiety Symptoms

We compared social anxiety symptoms between the experimental and waitlist groups. No significant difference was observed between the experimental and waitlist groups regarding social anxiety in either virtual or physical communities (Table 5). Thus, these results did not support hypothesis 2.

**Table 5.** Intention-to-treat analysis of latent growth curve modeling of social anxiety symptoms in the overall sample. The Gelman–Rubin statistics (rhat) for all coefficients was 1.0.

	Intervention effect					Baseline (Wave 1), mean (SD)	Wave 2, mean (SD)	Wave 3, mean (SD)	Wave 4, mean (SD)	SMD^a^
	β	95% LL^b^	95% UL^c^	Wald	*P* value					
Social anxiety symptoms in virtual communities^d,e^										
Experiment	0.666	–0.109	1.48	1.649	.099	28.95 (18.97)	31.89 (17.85)	31.87 (17.64)	31.96 (18.4)	0.11
Waitlist	—^f^	—	—	—	—	28.39 (19.07)	31.12 (18.04)	30.79 (17.87)	29.79 (18.92)	—
Social anxiety symptoms in physical communities^g^										
Experiment	0.319	–0.534	1.095	0.771	.441	46.81 (23.49)	48.93 (21.21)	47.79 (20.41)	47.61 (20.53)	0.06
Waitlist	—	—	—	—	—	45.66 (23.22)	46.24 (21.3)	46.07 (20.51)	45.54 (21.16)	—

a SMD: Standardized Mean Difference.

b LL: lower limit.

c UL: upper limit.

d Widely Applicable Bayesian Information Criterion (WAIC) -15995.2, leave-one-out (LOO) -16147.0.

e LOO -16467.5.

f Not applicable.

g WAIC -16332.1.

We likewise tested hypothesis 2 among participants with social anxiety disorder only (n=944; Modified LSAS-J≥30). As presented in Table 6, even when restricting the sample to individuals with social anxiety disorder, no statistically significant difference in social anxiety symptoms was observed between the experimental and waitlist control groups. This result suggests that the effectiveness of online game–based exposure therapy may be limited, irrespective of the severity level of social anxiety symptoms.

**Table 6.** Intention-to-treat analysis of latent growth curve modeling of social anxiety symptoms in individuals with social anxiety disorders^a^^,^^b^. The Gelman–Rubin statistics (rhat) for all coefficients was 1.0. The number of participants in the experiment and waitlist groups was 470 and 474, respectively.

	Intervention effect					Baseline (Wave 1), mean (SD)	Wave 2, mean (SD)	Wave 3, mean (SD)	Wave 4, mean (SD)	SMD^c^
	B	95% LL^d^	95% UL^e^	Wald	*P* value					
Social anxiety symptoms in virtual communities^a^										
Experiment	0.82	–0.05	1.68	1.83	.07	32.17 (18.02)	34.85 (17.05)	34.80 (16.80)	34.93 (17.59)	0.13
Waitlist	—^f^	—	—	—	—	32.07 (17.87)	33.97 (16.92)	33.68 (16.75)	32.97 (18.00)	—
Social anxiety symptoms in physical communities^b^										
Experiment	0.27	–0.61	1.12	0.61	.54	53.37 (18.31)	53.75 (18.12)	52.58 (17.22)	52.35 (17.50)	0.04
Waitlist	—	—	—	—	—	52.38 (18.00)	51.36 (17.83)	50.84 (17.43)	50.73 (17.92)	—

a Widely Applicable Bayesian Information Criterion (WAIC) -13643.1, leave-one-out (LOO) -13778.3.

b WAIC -13775.6, LOO -13895.1.

c SMD: standardized mean difference.

d LL: lower limit.

e UL: upper limit.

f Not applicable.

### Association Between Online Game Behaviors and Depressive Symptoms

**Figure 7.**
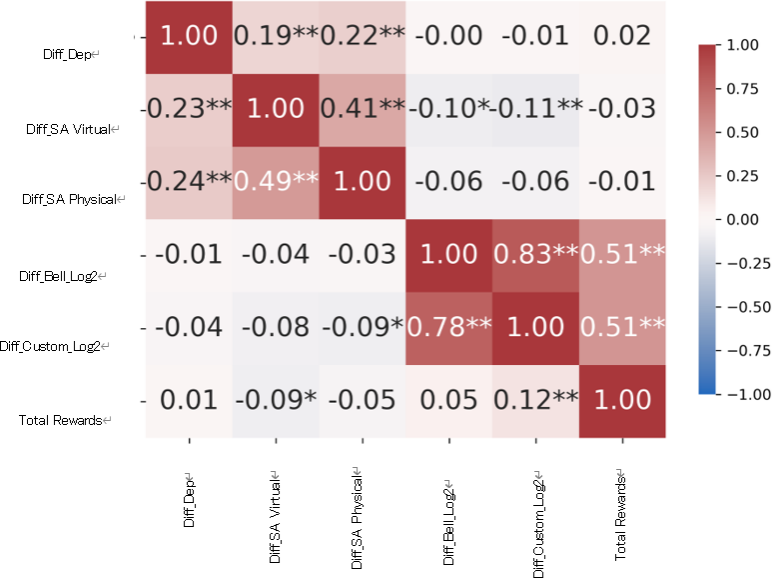
Correlation matrix among depressive symptoms, social anxiety, and online game behaviors. The upper half is the correlation coefficient for the experimental group, and the lower half is the correlation coefficient for the waitlist group. Diff indicates the change score calculated by subtracting the baseline value from the follow-up value; Dep denotes depression; SA denotes social anxiety; Bell indicates the number of times the bell was rung; Custom refers to the number of times the avatar’s clothing was customized; and Log2 denotes that the data were log-transformed using base 2.*P<.05, **P<.01.

The association between online game behaviors and depressive symptoms was examined through correlation analysis. In Figure 7, Diff_Dep represents the value obtained by subtracting depressive symptoms at wave 1 from those at wave 4. That is, a more negative value indicates improvement during the intervention period. Similarly, Diff_bell_countLOG2 is the difference in bell-ringing frequency (log2-transformed) between wave 4 and wave 1, and Diff_customl_countLOG2 is the difference in avatar customization frequency (log2-transformed) between the same waves. In addition, total reward indicates the number of bonus rewards received, ranging from 0 to 3.

The upper and lower halves of Figure 7 showed correlation matrices for the experimental and waitlist groups, respectively. No significant correlations were observed between online game behaviors and depressive symptoms in either group.

### Association Between Online Game Behaviors and Social Anxiety Symptoms

The association between online game behaviors and social anxiety symptoms was examined through correlation analysis. In Figure 7, Diff_SA_V represents the difference in virtual-community social anxiety symptoms in the Pigg Party between waves 4 and 1. Similarly, Diff_SA_P represents the difference in physical-community social anxiety symptoms between those waves. A more negative value indicates greater improvement during the intervention.

In the experimental group, a higher frequency of bell ringing was associated with a reduction in social anxiety symptoms (*r*=–0.10). Likewise, a higher frequency of avatar customization was associated with a reduction in virtual social anxiety symptoms (*r*=–0.11). Even in the waitlist group, a higher frequency of avatar customization was associated with a reduction in physical-community social anxiety symptoms (*r*=–0.09). Higher frequencies of receiving additional bonuses were also associated with greater improvement in virtual social anxiety symptoms (*r*=–0.09). However, the effect sizes of these correlation coefficients were all below the threshold for a small effect (0.2), and consistent results were not observed between the experimental and waitlist groups; therefore, these findings should be interpreted with caution.

## Discussion

### Principal Results

This study investigated whether mental health symptoms can be improved through interventions on MMOGs by conducting a clinical trial with over 1000 participants. Consistent with previous findings [2,5], our results confirm that depressive symptoms can be alleviated through interventions on MMOGs. Given the large user base of MMOGs, such interventions may significantly contribute to reducing depressive symptoms on a broad scale [1]. Additionally, since the intervention was integrated as part of the in-game tasks, users could potentially experience an improvement in depressive symptoms while enjoying the game. Previous studies have used specific applications to improve depressive symptoms, which imposed a certain burden on users in terms of the intervention [18-21]. In contrast, since the participants in this study engaged with an MMOG interface they were already familiar with, the burden on users was minimal. This suggests that MMOG-based interventions could serve as an effective and accessible approach to alleviate depressive symptoms without imposing additional burden [11].

Although depressive symptoms in the experimental group improved significantly, no significant correlation was observed between bell-ringing behaviors and depressive symptoms, unlike the results reported in face-to-face behavioral activation studies [13-16]. This finding implies that bell-ringing behaviors may act merely as triggers, whereas other in-game activities—such as mutual visits or increased time spent chatting—could have contributed to the improvement of depressive symptoms [5].

Furthermore, contrary to previous studies [24-27,39], this study did not find evidence supporting an improvement in social anxiety symptoms. One possible explanation is the insufficient frequency of avatar customization in this study. Research suggests that if users do not customize their avatars frequently enough, they may struggle to identify with their avatars [36]. When users fail to fully customize their avatars, the discrepancy between in-game behaviors and real-life behaviors may increase [58], potentially explaining why the experimental group did not experience significant reductions in social anxiety symptoms [37]. Therefore, the participants in the experimental group may also have found it difficult to identify with their avatars [6], which consequently made it challenging to achieve the full effects of exposure therapy [28-30]. Moreover, in the experimental group, some participants may have intentionally rung bells when no one was present to avoid being noticed by others. In such cases, exposure to attention-focused situations may not have been effectively implemented. It is necessary to provide exposure situations that are difficult to avoid.

### Future Work

Although this study did not identify specific in-game behaviors related to depressive symptoms, future studies could let users self-select enjoyable in-game activities, followed by an examination of whether such activities contribute to improvement in depressive symptoms [5]. Identifying these specific in-game behaviors is crucial for implementing behavioral activation in MMOGs [17]. Although the effect size was small, greater avatar customization was associated with reduced social anxiety in both MMOGs and physical communities, suggesting that promoting avatar customization may still offer benefits. To uniformly increase avatar customization frequency, it may be effective to limit interventions to new users, for whom previous customization frequency is zero. This condition may enhance the observable intervention effects. Future studies should explicitly test whether increasing avatar customization frequency reduces social anxiety symptoms.

### Limitations

This study had 7 limitations. First, despite the random assignment of participants, the experimental group had a significantly lower proportion of females compared to the waitlist group. The gender imbalance may have influenced the results. Second, the platform was limited to Pigg Party [59]. Given that immersion is a key factor in exposure therapy [32], further research is needed to determine whether the finding can be replicated in MMOGs with a virtual reality headset. Third, it was suggested that randomly distributed rewards contributed to a reduction in virtual social anxiety symptoms in the waitlist group only. Moreover, this effect was insufficiently controlled. Fourth, it is also possible that the generally low baseline scores for depressive symptoms led to insufficient observable intervention effects. Fifth, because this study did not include indicators beyond symptom severity—such as quality of life—clinical implications cannot be fully inferred. Sixth, associations between specific gaming behaviors and depressive symptoms were not confirmed, and the mechanism for improvement of depressive symptoms in MMOGs could not be explained. Seventh, this study included participants across a broad age range, which precluded classification into a single age cohort. Future studies should be conducted with narrower and clearly defined age groups.

### Conclusions

This study, with a randomized controlled trial with over 1000 participants, highlights the potential of MMOG-based interventions to alleviate depressive symptoms. Notably, the MMOG used in this study was developed for leisure purposes rather than for therapeutic purposes [59]. This suggests that users experienced improvements in depressive symptoms while enjoying a leisure activity [4]. Using MMOGs to improve mental health may help prevent depressive symptoms in various individuals by providing an accessible interface through online games [3,11].

## Supplementary material

10.2196/73734 Multimedia Appendix 1 Movie showing example of an avatar changing clothes.

10.2196/73734 Multimedia Appendix 2 Percentage of participants with social anxiety disorder at baseline.

10.2196/73734 Multimedia Appendix 3 Percentage of participants with depressive disorder at baseline.

10.2196/73734 Multimedia Appendix 4 Timetable of Questionnaire Response Period and Intervention Period.

10.2196/73734 Multimedia Appendix 5 Brief Liebowitz Social Anxiety Scale in the Japanese version.

10.2196/73734 Multimedia Appendix 6 Frequency distribution of bell ringing events at baseline: comparison between original and log-transformed data.

10.2196/73734 Multimedia Appendix 7 Frequency distribution of avatar customization at baseline: comparison between original and log-transformed data.

10.2196/73734 Checklist 1 CONSORT checklist.

## Abbreviations

Brief LSAS-J: Japanese version of the Brief Liebowitz Social Anxiety Scale
FIML: Full Information Maximum Likelihood
LGCM: Latent Growth Curve Modeling
LOO: Leave-One-Out
MMOG: massive multiplayer online game
QIDS-J: Quick Inventory of Depressive Symptomatology
SMD: standardized mean difference
WAIC: Widely Applicable Bayesian Information Criterion
