# Supplementary material for: Improvement of Game Users’ Depressive Symptoms via Behavioral Activation in a Massive Multiplayer Online Game: Randomized Controlled Trial
Source: JMIR Serious Games. 2025 Sep 24;13:e73734. doi: 10.2196/73734 (PMC12459738; doi:10.2196/73734)
Supplement: Multimedia Appendix 2 [file games-v13-e73734-s002.docx]

Multimedia Appendix 2.


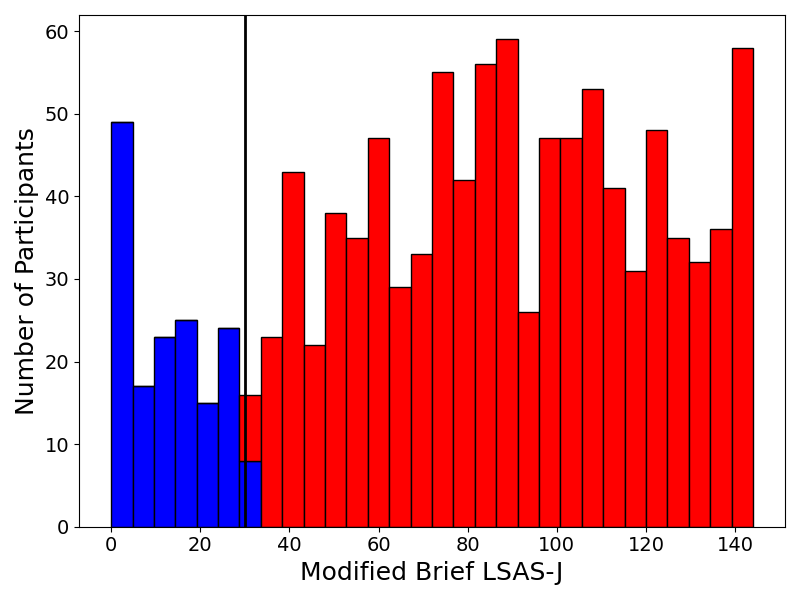


Adjusted Brief LSAS-J

*Notes:* The adjusted Brief LSAS-J (Brief Liebowitz Social Anxiety Scale in Japanese version) multiplies the 14-item score by 24/14 to ensure comparability with the 24-item original LSAS-J. Blue bars indicate the non-social anxiety group (score < 30; n = 161), and red bars indicate the social anxiety group (score ≥ 30; n = 944). The black vertical line represents the 30-point threshold.
