# Supplementary material for: Improvement of Game Users’ Depressive Symptoms via Behavioral Activation in a Massive Multiplayer Online Game: Randomized Controlled Trial
Source: JMIR Serious Games. 2025 Sep 24;13:e73734. doi: 10.2196/73734 (PMC12459738; doi:10.2196/73734)
Supplement: Multimedia Appendix 3 [file games-v13-e73734-s003.docx]

Multimedia Appendix 3.


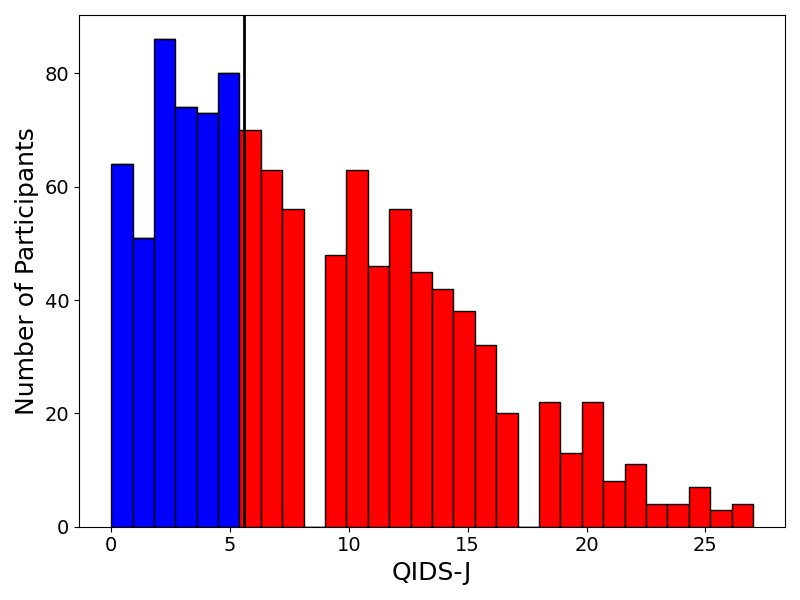


*Notes:* QIDS-J: Quick Inventory of Depressive Symptomatology, Japanese version. Blue bars represent the non-depression group (score < 6; n = 428), and red bars represent the depression group (score ≥ 6; n = 677). The black vertical line marks the 6-point threshold.
