# Supplementary material for: Improvement of Game Users’ Depressive Symptoms via Behavioral Activation in a Massive Multiplayer Online Game: Randomized Controlled Trial
Source: JMIR Serious Games. 2025 Sep 24;13:e73734. doi: 10.2196/73734 (PMC12459738; doi:10.2196/73734)
Supplement: Multimedia Appendix 4 [file games-v13-e73734-s004.docx]

Multimedia Appendix 4.

| Questionnaire response period | Intervention period |
| --- | --- |
|  |  |
| From 05 December to 15 December 2023 |  |
|  | From 16 December 2023 to 15 January 2024 |
|  |  |
|  |  |
| From 16 January to 26 January 2024 | From 16 January to 15 February 2024 |
|  |  |
|  |  |
| From 16 February to 26 February 2024 | From 16 February to 15 March 2024 |
|  |  |
|  |  |
| From 16 March to 26 March 2024 |  |
