# Supplementary material for: Improvement of Game Users’ Depressive Symptoms via Behavioral Activation in a Massive Multiplayer Online Game: Randomized Controlled Trial
Source: JMIR Serious Games. 2025 Sep 24;13:e73734. doi: 10.2196/73734 (PMC12459738; doi:10.2196/73734)
Supplement: Multimedia Appendix 5 [file games-v13-e73734-s005.docx]

Multimedia Appendix 5.

**Social anxiety scales.** To measure social anxiety in physical and virtual communities, we used a questionnaire that picked up 14 of the 24 items of the Liebowitz Social Anxiety Scale (LSAS) [1]. These questions correspond to items 2, 5–7, 10–12, 14–16, 18, 20, 21, and 23, respectively, on the Japanese version of the LSAS [2]. The validity of the modified version of this questionnaire has been established in our previous study [3].

To measure social anxiety in virtual communities (i.e., Pigg Party), the following instructions were presented to the participants.

*This measure assesses the role of social phobia in your life across situations in Pigg Party. Read each situation carefully and answer two questions about it; the first question asks about the level of anxiety or fear that you feel in the situation; the second question asks how often one should avoid it. If you come across a situation that you ordinarily do not experience, imagine “what if you were faced with that situation” and rate the degree to which you would fear this hypothetical situation and how frequently you would tend to avoid it (using the scale from 0 to 3 below). Please base your ratings on the influence of these situations on you in the last week.*

*fear or anxiety: none (0), mild (1), moderate (2), and severe (3)*

*avoidance: never (0%), occasionally (1–33%), often (33–67%), and usually (67–100%).*

*1. Participating in a small group activity in Pigg Party*

*2. Talking to a popular person in Pigg Party*

*3. Acting, performing, or speaking in front of an audience in Pigg Party*

*4. Going to a party in Pigg Party*

*5. Talking privately to someone you don’t know very well in Pigg Party*

*6. Talking face-to-face with someone you don’t know very well in Pigg Party*

*7. Meeting strangers in Pigg Party*

*8. Entering a room when others are already seated in Pigg Party*

*9. Being the center of attention in Pigg Party*

*10. Speaking up at a meeting in Pigg Party*

*11. Expressing disagreement with or disapproval of someone you don’t know very well in Pigg Party*

*12. Giving a prepared oral talk to a group in Pigg Party*

*13. Trying to make someone’s acquaintance for the purpose of a romantic/sexual relationship in Pigg Party*

*14. Hosting a party in Pigg Party*

To measure social anxiety in the physical community, the following instructions were presented to the participants.

*This measure assesses the role of social phobia in your life across scenarios in the physical community. Read each situation carefully and answer two questions about it; the first question asks the level of anxiety or fear you feel in a situation; the second question asks the frequency with which one should avoid it. If you encounter a situation that you ordinarily do not experience, imagine “what if you were faced with that situation” and then rate the degree to which you would fear this hypothetical situation and how often you would tend to avoid it (using the scale from 0 to 3 below). Please base your ratings on the effect of such situations on you in the last week.*

*fear or anxiety: none (0), mild (1), moderate (2), and severe (3)*

*avoidance: never (0%), occasionally (1%–33%), often (33%–67%), and usually (67%–100%).*

*1. Participating in a small group activity*

*2. Talking to a popular person*

*3. Acting, performing, or speaking in front of an audience*

*4. Going to a party*

*5. Calling someone you don’t know very well*

*6. Talking face-to-face with someone you don’t know very well*

*7. Meeting strangers*

*8. Entering a room when others are already seated*

*9. Being the center of attention*

*10. Speaking up in a meeting*

*11. Expressing disagreement with or disapproval of someone you don't know very well*

*12. Giving a prepared oral talk to a group*

*13. Trying to make someone’s acquaintance for the purpose of a romantic/sexual relationship*

*14. Hosting a party*

References

1. Heimberg RG, Horner KJ, Juster HR, Safren SA, Brown EJ, Schneier FR, Liebowitz MR. Psychometric properties of the Liebowitz Social Anxiety Scale. Psychological Medicine Cambridge University Press; 1999 Jan;29(1):199–212. doi: 10.1017/S0033291798007879

2. Asakura S, Inoue S, Sasaki F, Sasaki Y, Kitagawa N, Inoue T, Denda K, Koyama T, Ito M, Matsubara R. Reliability and validity of the Japanese version of the Liebowitz Social Anxiety Scale. Seishin Igaku Japan: Igaku Shoin; 2002;44(10):1077–1084.

3. Yokotani K, Takano M, Abe N, Kato TA. Improving social anxiety in lesbian, gay, bisexual, transgender, questioning, intersex and asexual individuals through avatar customization and communication. Asian Journal of Social Psychology 2025;28(1):e12666. doi: 10.1111/ajsp.12666
