# Supplementary material for: Improvement of Game Users’ Depressive Symptoms via Behavioral Activation in a Massive Multiplayer Online Game: Randomized Controlled Trial
Source: JMIR Serious Games. 2025 Sep 24;13:e73734. doi: 10.2196/73734 (PMC12459738; doi:10.2196/73734)
Supplement: Multimedia Appendix 7 [file games-v13-e73734-s007.docx]

Multimedia Appendix 7.


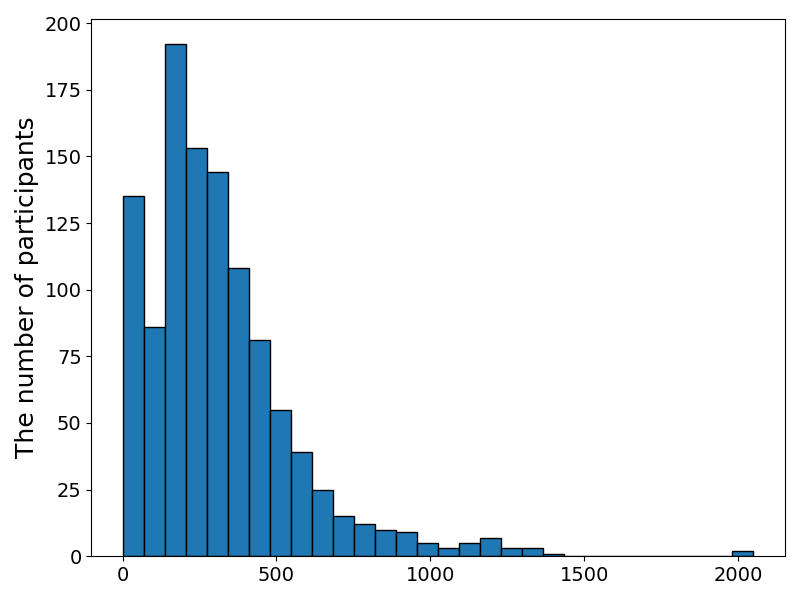


**Number of times the user customized own avatar at baseline**


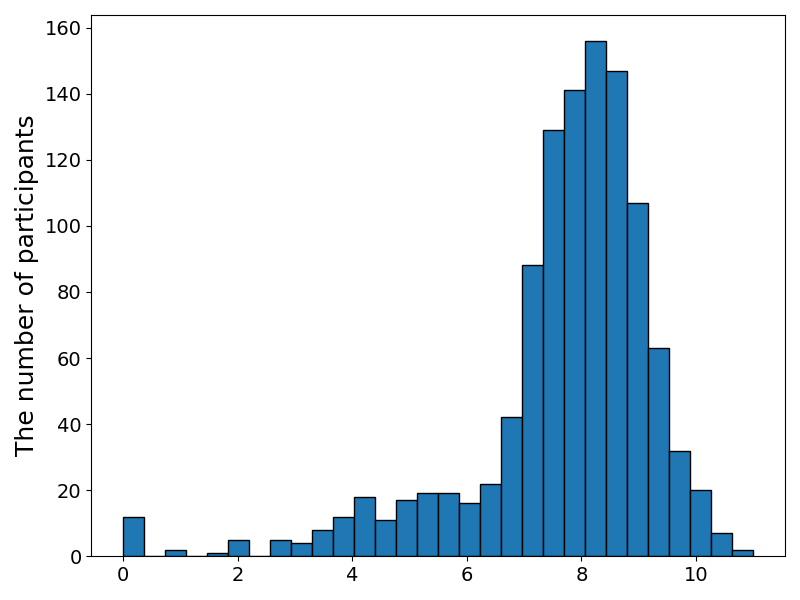


**Number of times the user customized own avatar at baseline (log-transformed)**
