# Supplementary material for: Improvement of Game Users’ Depressive Symptoms via Behavioral Activation in a Massive Multiplayer Online Game: Randomized Controlled Trial
Source: JMIR Serious Games. 2025 Sep 24;13:e73734. doi: 10.2196/73734 (PMC12459738; doi:10.2196/73734)
Supplement: Checklist 1 [file games-v13-e73734-s008.pdf]

CONSORT-EHEALTH (V 1.6.1) - Submission/Publication Form

The CONSORT-EHEALTH checklist is intended for authors of randomized trials evaluating web-based and Internet-based applications/interventions, including mobile interventions, electronic games (incl multiplayer games), social media, certain telehealth applications, and other interactive and/or networked electronic applications. Some of the items (e.g. all subitems under item 5 - description of the intervention) may also be applicable for other study designs.

The goal of the CONSORT EHEALTH checklist and guideline is to be
a) a guide for reporting for authors of RCTs,
b) to form a basis for appraisal of an ehealth trial (in terms of validity)

CONSORT-EHEALTH items/subitems are MANDATORY reporting items for studies published in the Journal of Medical Internet Research and other journals / scientific societies endorsing the checklist.

Items numbered 1., 2., 3., 4a., 4b etc are original CONSORT or CONSORT-NPT (non-pharmacologic treatment) items.
Items with Roman numerals (i., ii, iii, iv etc.) are CONSORT-EHEALTH extensions/clarifications.

As the CONSORT-EHEALTH checklist is still considered in a formative stage, we would ask that you also RATE ON A SCALE OF 1-5 how important/useful you feel each item is FOR THE PURPOSE OF THE CHECKLIST and reporting guideline (optional).

Mandatory reporting items are marked with a red *.
In the textboxes, either copy & paste the relevant sections from your manuscript into this form - please include any quotes from your manuscript in QUOTATION MARKS,
or answer directly by providing additional information not in the manuscript, or elaborating on why the item was not relevant for this study.

YOUR ANSWERS WILL BE PUBLISHED AS A SUPPLEMENTARY FILE TO YOUR PUBLICATION IN JMIR AND ARE CONSIDERED PART OF YOUR PUBLICATION (IF ACCEPTED).
Please fill in these questions diligently. Information will not be copyedited, so please use proper spelling and grammar, use correct capitalization, and avoid abbreviations.

DO NOT FORGET TO SAVE AS PDF _AND_ CLICK THE SUBMIT BUTTON SO YOUR ANSWERS ARE IN OUR DATABASE !!!

Citation Suggestion (if you append the pdf as Appendix we suggest to cite this paper in the caption):
Eysenbach G, CONSORT-EHEALTH Group
CONSORT-EHEALTH: Improving and Standardizing Evaluation Reports of Web-based and Mobile Health Interventions
J Med Internet Res 2011;13(4):e126

URL: http://www.jmir.org/2011/4/e126/
doi: 10.2196/jmir.1923
PMID: 22209829

Google にログインすると作業内容を保存できます。詳細

* 必須の質問です

Your name *

First Last

Kenji Yokotani

Primary Affiliation (short), City, Country *

University of Toronto, Toronto, Canada

Tokushima University, Tokushima, Japan

Your e-mail address *

abc@gmail.com

yokotanikenji@tokushima-u.ac.jp

Title of your manuscript *

Provide the (draft) title of your manuscript.

Behavioral Activation in a Massive Multiplayer Online Game Improves Game Users' Depressive Symptoms: A Randomized Controlled Trial

Name of your App/Software/Intervention *

If there is a short and a long/alternate name, write the short name first and add the long name in brackets.

Pigg Party

Evaluated Version (if any)

e.g. "V1", "Release 2017-03-01", "Version 2.0.27913"

回答を入力

Language(s) *

What language is the intervention/app in? If multiple languages are available, separate by comma (e.g. "English, French")

Japanese

URL of your Intervention Website or App

e.g. a direct link to the mobile app on app in appstore (itunes, Google Play), or URL of the website. If the intervention is a DVD or hardware, you can also link to an Amazon page.

https://play.google.com/store/apps/details?id=jp.co.cyberagent.miami&hl=ja

URL of an image/screenshot (optional)

回答を入力

Accessibility *

Can an enduser access the intervention presently?

- (selected) access is free and open
- access only for special usergroups, not open
- access is open to everyone, but requires payment/subscription/in-app purchases
- app/intervention no longer accessible
- その他:

Primary Medical Indication/Disease/Condition *

e.g. "Stress", "Diabetes", or define the target group in brackets after the condition, e.g. "Autism (Parents of children with)", "Alzheimers (Informal Caregivers of)"

Pigg Party users(Non clinical group)

Primary Outcomes measured in trial *

comma-separated list of primary outcomes reported in the trial

Depression, Social Anxiety

Secondary/other outcomes

Are there any other outcomes the intervention is expected to affect?

Frequency of ringing a friend's doorbell in the Pigg Party

Recommended "Dose" *

What do the instructions for users say on how often the app should be used?

- (●) Approximately Daily
- ( ) Approximately Weekly
- ( ) Approximately Monthly
- ( ) Approximately Yearly
- ( ) "as needed"
- ( ) その他:

Approx. Percentage of Users (starters) still using the app as recommended after 3 months *

- (●) unknown / not evaluated
- ( ) 0-10%
- ( ) 11-20%
- ( ) 21-30%
- ( ) 31-40%
- ( ) 41-50%
- ( ) 51-60%
- ( ) 61-70%
- ( ) 71%-80%
- ( ) 81-90%
- ( ) 91-100%
- ( ) その他:

Overall, was the app/intervention effective? *

- ○ yes: all primary outcomes were significantly better in intervention group vs control
- ◉ partly: SOME primary outcomes were significantly better in intervention group vs control
- ○ no statistically significant difference between control and intervention
- ○ potentially harmful: control was significantly better than intervention in one or more outcomes
- ○ inconclusive: more research is needed
- ○ その他:

Article Preparation Status/Stage *

At which stage in your article preparation are you currently (at the time you fill in this form)

- ○ not submitted yet - in early draft status
- ○ not submitted yet - in late draft status, just before submission
- ◉ submitted to a journal but not reviewed yet
- ○ submitted to a journal and after receiving initial reviewer comments
- ○ submitted to a journal and accepted, but not published yet
- ○ published
- ○ その他:

Journal *

If you already know where you will submit this paper (or if it is already submitted), please provide the journal name (if it is not JMIR, provide the journal name under "other")

- ○ not submitted yet / unclear where I will submit this
- ○ Journal of Medical Internet Research (JMIR)
- ○ JMIR mHealth and UHealth
- ◉ JMIR Serious Games
- ○ JMIR Mental Health
- ○ JMIR Public Health
- ○ JMIR Formative Research
- ○ Other JMIR sister journal
- ○ その他:

Is this a full powered effectiveness trial or a pilot/feasibility trial? *

- ○ Pilot/feasibility
- ◉ Fully powered

Manuscript tracking number *

If this is a JMIR submission, please provide the manuscript tracking number under "other" (The ms tracking number can be found in the submission acknowledgement email, or when you login as author in JMIR. If the paper is already published in JMIR, then the ms tracking number is the four-digit number at the end of the DOI, to be found at the bottom of each published article in JMIR)

- ○ no ms number (yet) / not (yet) submitted to / published in JMIR
- ◉ その他: #73734

TITLE AND ABSTRACT

1a) TITLE: Identification as a randomized trial in the title

1a) Does your paper address CONSORT item 1a? *

I.e does the title contain the phrase "Randomized Controlled Trial"? (if not, explain the reason under "other")

- (●) yes
- ( ) その他:

1a-i) Identify the mode of delivery in the title

Identify the mode of delivery. Preferably use “web-based” and/or “mobile” and/or “electronic game” in the title. Avoid ambiguous terms like “online”, “virtual”, “interactive”. Use “Internet-based” only if Intervention includes non-web-based Internet components (e.g. email), use “computer-based” or “electronic” only if offline products are used. Use “virtual” only in the context of “virtual reality” (3-D worlds). Use “online” only in the context of “online support groups”. Complement or substitute product names with broader terms for the class of products (such as “mobile” or “smart phone” instead of “iphone”), especially if the application runs on different platforms.

	1	2	3	4	5	
subitem not at all important	○	○	○	○	●	essential

選択を解除

Does your paper address subitem 1a-i? *

Copy and paste relevant sections from manuscript title (include quotes in quotation marks "like this" to indicate direct quotes from your manuscript), or elaborate on this item by providing additional information not in the ms, or briefly explain why the item is not applicable/relevant for your study

Massive Multiplayer Online Game

1a-ii) Non-web-based components or important co-interventions in title

Mention non-web-based components or important co-interventions in title, if any (e.g., “with telephone support”).

	1	2	3	4	5	
subitem not at all important	◉	○	○	○	○	essential

選択を解除

Does your paper address subitem 1a-ii?

Copy and paste relevant sections from manuscript title (include quotes in quotation marks "like this" to indicate direct quotes from your manuscript), or elaborate on this item by providing additional information not in the ms, or briefly explain why the item is not applicable/relevant for your study

Since that element is not present in this experiment, it is not entered in the title.

1a-iii) Primary condition or target group in the title

Mention primary condition or target group in the title, if any (e.g., “for children with Type I Diabetes”) Example: A Web-based and Mobile Intervention with Telephone Support for Children with Type I Diabetes: Randomized Controlled Trial

	1	2	3	4	5	
subitem not at all important	○	○	○	○	◉	essential

選択を解除

Does your paper address subitem 1a-iii? *

Copy and paste relevant sections from manuscript title (include quotes in quotation marks "like this" to indicate direct quotes from your manuscript), or elaborate on this item by providing additional information not in the ms, or briefly explain why the item is not applicable/relevant for your study

Behavioral Activation in a Massive Multiplayer Online Game Improves Game Users’ Depressive Symptoms: A Randomized Controlled Trial

1b) ABSTRACT: Structured summary of trial design, methods, results, and conclusions

NPT extension: Description of experimental treatment, comparator, care providers, centers, and blinding status.

1b-i) Key features/functionalities/components of the intervention and comparator in the METHODS section of the ABSTRACT

Mention key features/functionalities/components of the intervention and comparator in the abstract. If possible, also mention theories and principles used for designing the site. Keep in mind the needs of systematic reviewers and indexers by including important synonyms. (Note: Only report in the abstract what the main paper is reporting. If this information is missing from the main body of text, consider adding it)

	1	2	3	4	5	
subitem not at all important	○	○	○	○	◉	essential

選択を解除

Does your paper address subitem 1b-i? *

Copy and paste relevant sections from the manuscript abstract (include quotes in quotation marks "like this" to indicate direct quotes from your manuscript), or elaborate on this item by providing additional information not in the ms, or briefly explain why the item is not applicable/relevant for your study

Background: Online games developed to improve mental health symptoms are reportedly effective among game users. However, it has not been verified whether massive multiplayer online games (MMOGs) developed for leisure purposes are effective in improving users' mental health symptoms. Objective: Hence, with the theoretical background of behavioral activation, in which increasing behavior tied to rewards reduces depressive symptoms, we aimed to examine whether interventions on the MMOG could improve users' depressive and social anxiety symptoms using a Japanese MMOG called Pigg Party.

1b-ii) Level of human involvement in the METHODS section of the ABSTRACT

Clarify the level of human involvement in the abstract, e.g., use phrases like “fully automated” vs. “therapist/nurse/care provider/physician-assisted” (mention number and expertise of providers involved, if any). (Note: Only report in the abstract what the main paper is reporting. If this information is missing from the main body of text, consider adding it)

	1	2	3	4	5	
subitem not at all important	(●)	○	○	○	○	essential

選択を解除

Does your paper address subitem 1b-ii?

Copy and paste relevant sections from the manuscript abstract (include quotes in quotation marks "like this" to indicate direct quotes from your manuscript), or elaborate on this item by providing additional information not in the ms, or briefly explain why the item is not applicable/relevant for your study

Since that element is not present in this experiment, it is not entered in the abstract.

1b-iii) Open vs. closed, web-based (self-assessment) vs. face-to-face assessments in the METHODS section of the ABSTRACT

Mention how participants were recruited (online vs. offline), e.g., from an open access website or from a clinic or a closed online user group (closed usergroup trial), and clarify if this was a purely web-based trial, or there were face-to-face components (as part of the intervention or for assessment). Clearly say if outcomes were self-assessed through questionnaires (as common in web-based trials). Note: In traditional offline trials, an open trial (open-label trial) is a type of clinical trial in which both the researchers and participants know which treatment is being administered. To avoid confusion, use “blinded” or “unblinded” to indicated the level of blinding instead of “open”, as “open” in web-based trials usually refers to “open access” (i.e. participants can self-enrol). (Note: Only report in the abstract what the main paper is reporting. If this information is missing from the main body of text, consider adding it)

	1	2	3	4	5	
subitem not at all important	○	○	○	○	(●)	essential

選択を解除

Does your paper address subitem 1b-iii?

Copy and paste relevant sections from the manuscript abstract (include quotes in quotation marks "like this" to indicate direct quotes from your manuscript), or elaborate on this item by providing additional information not in the ms, or briefly explain why the item is not applicable/relevant for your study

Depressive and social anxiety symptoms were measured using questionnaires at the start of the experiment and at one, two, and three months later.

1b-iv) RESULTS section in abstract must contain use data

Report number of participants enrolled/assessed in each group, the use/uptake of the intervention (e.g., attrition/adherence metrics, use over time, number of logins etc.), in addition to primary/secondary outcomes. (Note: Only report in the abstract what the main paper is reporting. If this information is missing from the main body of text, consider adding it)

	1	2	3	4	5	
subitem not at all important	○	○	○	○	◉	essential

選択を解除

Does your paper address subitem 1b-iv?

Copy and paste relevant sections from the manuscript abstract (include quotes in quotation marks "like this" to indicate direct quotes from your manuscript), or elaborate on this item by providing additional information not in the ms, or briefly explain why the item is not applicable/relevant for your study

Participants were 1,105 users of the Pigg Party, and they were randomly assigned to an experimental group and a waitlist group.

1b-v) CONCLUSIONS/DISCUSSION in abstract for negative trials

Conclusions/Discussions in abstract for negative trials: Discuss the primary outcome - if the trial is negative (primary outcome not changed), and the intervention was not used, discuss whether negative results are attributable to lack of uptake and discuss reasons. (Note: Only report in the abstract what the main paper is reporting. If this information is missing from the main body of text, consider adding it)

	1	2	3	4	5	
subitem not at all important	◉	○	○	○	○	essential

選択を解除

Does your paper address subitem 1b-v?

Copy and paste relevant sections from the manuscript abstract (include quotes in quotation marks "like this" to indicate direct quotes from your manuscript), or elaborate on this item by providing additional information not in the ms, or briefly explain why the item is not applicable/relevant for your study

Since that element is not present in this experiment, it is not entered in the abstract.

INTRODUCTION

2a) In INTRODUCTION: Scientific background and explanation of rationale

2a-i) Problem and the type of system/solution

Describe the problem and the type of system/solution that is object of the study: intended as stand-alone intervention vs. incorporated in broader health care program? Intended for a particular patient population? Goals of the intervention, e.g., being more cost-effective to other interventions, replace or complement other solutions? (Note: Details about the intervention are provided in “Methods” under 5)

	1	2	3	4	5	
subitem not at all important	○	○	○	○	◉	essential

選択を解除

Does your paper address subitem 2a-i? *

Copy and paste relevant sections from the manuscript (include quotes in quotation marks "like this" to indicate direct quotes from your manuscript), or elaborate on this item by providing additional information not in the ms, or briefly explain why the item is not applicable/relevant for your study

Interventions using online games to improve the mental health symptoms of game users have been increasingly implemented in recent years[1]. These online games reportedly improve depression[2] and anxiety[3] symptoms. Although most of these online games have been developed to improve mental health symptoms, massive multiplayer online games (MMOGs) developed for leisure purposes[4] might have similar effects on depressive[5] and anxiety symptoms[6]. However, as few studies have examined the intervention effects of MMOGs on depressive and anxiety symptoms using clinical trial designs[5], their efficacy remains unclear. Previous studies has not adequately answered whether MMOGs improve users’ depressive and anxiety symptoms. This study aimed to investigate this issue.

2a-ii) Scientific background, rationale: What is known about the (type of) system

Scientific background, rationale: What is known about the (type of) system that is the object of the study (be sure to discuss the use of similar systems for other conditions/diagnoses, if appropiate), motivation for the study, i.e. what are the reasons for and what is the context for this specific study, from which stakeholder viewpoint is the study performed, potential impact of findings [2]. Briefly justify the choice of the comparator.

	1	2	3	4	5	
subitem not at all important	○	○	○	○	◉	essential

選択を解除

Does your paper address subitem 2a-ii? *

Copy and paste relevant sections from the manuscript (include quotes in quotation marks "like this" to indicate direct quotes from your manuscript), or elaborate on this item by providing additional information not in the ms, or briefly explain why the item is not applicable/relevant for your study

This study employed two theoretical frameworks. First is the behavioral activation of depressive symptoms[7]. Individuals with depressive symptoms have difficulty learning the association between their behaviors and the rewards linked to them[8], which makes it challenging for them to experience positive emotional states and facilitates the maintenance of these symptoms[9]. By increasing behaviors associated with rewards, they can learn reward-associated behaviors, experience positive emotional states, and improve depressive symptoms[10]. Meta-analysis of behavioral activation shows that activation of reward-associated behaviors improves depressive symptoms[11]. Activation of reward-associated behaviors in the physical community—implemented through online games, such as web page applications [12], short message services[13], and smartphone applications[14]—has demonstrated efficacy in improving depressive symptoms. Activation of reward-associated behaviors within online games also improved users' depressive symptoms[15]. These studies suggest that the activation of reward-associated behaviors in MMOGs improves the depressive symptoms of game users[5].

2b) In INTRODUCTION: Specific objectives or hypotheses

Does your paper address CONSORT subitem 2b? *

Copy and paste relevant sections from the manuscript (include quotes in quotation marks "like this" to indicate direct quotes from your manuscript), or elaborate on this item by providing additional information not in the ms, or briefly explain why the item is not applicable/relevant for your study

Our hypotheses are as follows: (1) according to behavioral activation on depressive symptoms[7,15], the experimental group would exhibit improvements in depressive symptoms compared to the waitlist group. (2) According to exposure therapy on social anxiety symptoms[16,21], the experimental group would exhibit improvements in social anxiety, both within an MMOG and in the physical community, compared to that of the waitlist group.

METHODS

3a) Description of trial design (such as parallel, factorial) including allocation ratio

Does your paper address CONSORT subitem 3a? *

Copy and paste relevant sections from the manuscript (include quotes in quotation marks "like this" to indicate direct quotes from your manuscript), or elaborate on this item by providing additional information not in the ms, or briefly explain why the item is not applicable/relevant for your study

This study involved a single-site, randomized, parallel-group comparative trial (Trial Registration: WDB9U). The study protocol was approved by the Ethics Committee of the National University A in Japan. The detailed protocol for this experiment has been disclosed previously[30].

3b) Important changes to methods after trial commencement (such as eligibility criteria), with reasons

Does your paper address CONSORT subitem 3b? *

Copy and paste relevant sections from the manuscript (include quotes in quotation marks "like this" to indicate direct quotes from your manuscript), or elaborate on this item by providing additional information not in the ms, or briefly explain why the item is not applicable/relevant for your study

Since that element is not present in this experiment, it is not entered in the method.

3b-i) Bug fixes, Downtimes, Content Changes

Bug fixes, Downtimes, Content Changes: ehealth systems are often dynamic systems. A description of changes to methods therefore also includes important changes made on the intervention or comparator during the trial (e.g., major bug fixes or changes in the functionality or content) (5-iii) and other “unexpected events” that may have influenced study design such as staff changes, system failures/downtimes, etc. [2].

	1	2	3	4	5	
subitem not at all important	(●)	○	○	○	○	essential

選択を解除

Does your paper address subitem 3b-i?

Copy and paste relevant sections from the manuscript (include quotes in quotation marks "like this" to indicate direct quotes from your manuscript), or elaborate on this item by providing additional information not in the ms, or briefly explain why the item is not applicable/relevant for your study

Since that element is not present in this experiment, it is not entered in the method.

4a) Eligibility criteria for participants

Does your paper address CONSORT subitem 4a? *

Copy and paste relevant sections from the manuscript (include quotes in quotation marks "like this" to indicate direct quotes from your manuscript), or elaborate on this item by providing additional information not in the ms, or briefly explain why the item is not applicable/relevant for your study

The participants of this study were users who had been using the Pigg Party before September 15, 2023. On December 5, 2023, the administrator of the Pigg Party emailed the users requesting their participation in the study. By December 15, 2023, 1,331 users had expressed their willingness to participate, and then their eligibility for participation was assessed. As a result, 226 users were removed from the participants; among these, 175 users had withdrawn their consent to participate in the experiment, and after the recruitment period, 51 users requested to participate. The final participants consisted of 1,105 Pigg Party users.

4a-i) Computer / Internet literacy

Computer / Internet literacy is often an implicit “de facto” eligibility criterion - this should be explicitly clarified.

	1	2	3	4	5	
subitem not at all important	○	○	○	○	◉	essential

選択を解除

Does your paper address subitem 4a-i?

Copy and paste relevant sections from the manuscript (include quotes in quotation marks "like this" to indicate direct quotes from your manuscript), or elaborate on this item by providing additional information not in the ms, or briefly explain why the item is not applicable/relevant for your study

The participants of this study were users who had been using the Pigg Party before September 15, 2023. On December 5, 2023, the administrator of the Pigg Party emailed the users requesting their participation in the study. By December 15, 2023, 1,331 users had expressed their willingness to participate, and then their eligibility for participation was assessed. As a result, 226 users were removed from the participants; among these, 175 users had withdrawn their consent to participate in the experiment, and after the recruitment period, 51 users requested to participate. The final participants consisted of 1,105 Pigg Party users.

4a-ii) Open vs. closed, web-based vs. face-to-face assessments:

Open vs. closed, web-based vs. face-to-face assessments: Mention how participants were recruited (online vs. offline), e.g., from an open access website or from a clinic, and clarify if this was a purely web-based trial, or there were face-to-face components (as part of the intervention or for assessment), i.e., to what degree got the study team to know the participant. In online-only trials, clarify if participants were quasi-anonymous and whether having multiple identities was possible or whether technical or logistical measures (e.g., cookies, email confirmation, phone calls) were used to detect/prevent these.

	1	2	3	4	5	
subitem not at all important	○	○	○	○	◉	essential

選択を解除

Does your paper address subitem 4a-ii? *

Copy and paste relevant sections from the manuscript (include quotes in quotation marks "like this" to indicate direct quotes from your manuscript), or elaborate on this item by providing additional information not in the ms, or briefly explain why the item is not applicable/relevant for your study

The participants of this study were users who had been using the Pigg Party before September 15, 2023. On December 5, 2023, the administrator of the Pigg Party emailed the users requesting their participation in the study. By December 15, 2023, 1,331 users had expressed their willingness to participate, and then their eligibility for participation was assessed. As a result, 226 users were removed from the participants; among these, 175 users had withdrawn their consent to participate in the experiment, and after the recruitment period, 51 users requested to participate. The final participants consisted of 1,105 Pigg Party users.

4a-iii) Information giving during recruitment

Information given during recruitment. Specify how participants were briefed for recruitment and in the informed consent procedures (e.g., publish the informed consent documentation as appendix, see also item X26), as this information may have an effect on user self-selection, user expectation and may also bias results.

	1	2	3	4	5	
subitem not at all important	○	○	○	○	◉	essential

選択を解除

Does your paper address subitem 4a-iii?

Copy and paste relevant sections from the manuscript (include quotes in quotation marks "like this" to indicate direct quotes from your manuscript), or elaborate on this item by providing additional information not in the ms, or briefly explain why the item is not applicable/relevant for your study

The participants of this study were users who had been using the Pigg Party before September 15, 2023. On December 5, 2023, the administrator of the Pigg Party emailed the users requesting their participation in the study. By December 15, 2023, 1,331 users had expressed their willingness to participate, and then their eligibility for participation was assessed. As a result, 226 users were removed from the participants; among these, 175 users had withdrawn their consent to participate in the experiment, and after the recruitment period, 51 users requested to participate. The final participants consisted of 1,105 Pigg Party users.

4b) Settings and locations where the data were collected

Does your paper address CONSORT subitem 4b? *

Copy and paste relevant sections from the manuscript (include quotes in quotation marks "like this" to indicate direct quotes from your manuscript), or elaborate on this item by providing additional information not in the ms, or briefly explain why the item is not applicable/relevant for your study

Design
This study involved a single-site, randomized, parallel-group comparative trial (Trial Registration: WDB9U). The study protocol was approved by the Ethics Committee of the National University A in Japan. The detailed protocol for this experiment has been disclosed previously[30].

4b-i) Report if outcomes were (self-)assessed through online questionnaires

Clearly report if outcomes were (self-)assessed through online questionnaires (as common in web-based trials) or otherwise.

	1	2	3	4	5	
subitem not at all important	○	○	○	○	◉	essential

選択を解除

Does your paper address subitem 4b-i? *

Copy and paste relevant sections from the manuscript (include quotes in quotation marks "like this" to indicate direct quotes from your manuscript), or elaborate on this item by providing additional information not in the ms, or briefly explain why the item is not applicable/relevant for your study

To assess depressive symptoms, the Quick Inventory of Depressive Symptomatology in Japanese version (QIDS-J) was employed[32,33]. The QIDS-J is a 16-item questionnaire that uses a 4-point Likert scale, with higher scores indicating greater severity of depression. The Cronbach's alpha coefficient for the QIDS-J at baseline was .86.

To assess social anxiety symptoms, the Brief Liebowitz Social Anxiety Scale in Japanese version (Brief LSAS-J) was used[6,34]. The Brief LSAS-J can measure social anxiety symptoms in both virtual and physical communities. It consists of a 14-item questionnaire for each community that assesses anxiety symptoms and avoidance behaviors. Higher scores on the Brief LSAS-J indicate greater severity of social anxiety symptoms in both virtual and physical communities. The Cronbach's alpha coefficients for the Brief LSAS-J at baseline in virtual and physical communities were .95 and .97, respectively.

4b-ii) Report how institutional affiliations are displayed

Report how institutional affiliations are displayed to potential participants [on ehealth media], as affiliations with prestigious hospitals or universities may affect volunteer rates, use, and reactions with regards to an intervention.(Not a required item – describe only if this may bias results)

	1	2	3	4	5	
subitem not at all important	◉	○	○	○	○	essential

選択を解除

Does your paper address subitem 4b-ii?

Copy and paste relevant sections from the manuscript (include quotes in quotation marks "like this" to indicate direct quotes from your manuscript), or elaborate on this item by providing additional information not in the ms, or briefly explain why the item is not applicable/relevant for your study

Since that element is not present in this experiment, it is not entered in the method.

5) The interventions for each group with sufficient details to allow replication, including how and when they were actually administered

5-i) Mention names, credential, affiliations of the developers, sponsors, and owners

Mention names, credential, affiliations of the developers, sponsors, and owners [6] (if authors/evaluators are owners or developer of the software, this needs to be declared in a “Conflict of interest” section or mentioned elsewhere in the manuscript).

	1	2	3	4	5	
subitem not at all important	○	○	○	○	◉	essential

選択を解除

Does your paper address subitem 5-i?

Copy and paste relevant sections from the manuscript (include quotes in quotation marks "like this" to indicate direct quotes from your manuscript), or elaborate on this item by providing additional information not in the ms, or briefly explain why the item is not applicable/relevant for your study

Kenji Yokotani was funded by CyberAgent, Inc. Masanori Takano was an employee of CyberAgent, Inc.

5-ii) Describe the history/development process

Describe the history/development process of the application and previous formative evaluations (e.g., focus groups, usability testing), as these will have an impact on adoption/use rates and help with interpreting results.

	1	2	3	4	5	
subitem not at all important	○	○	●	○	○	essential

選択を解除

Does your paper address subitem 5-ii?

Copy and paste relevant sections from the manuscript (include quotes in quotation marks "like this" to indicate direct quotes from your manuscript), or elaborate on this item by providing additional information not in the ms, or briefly explain why the item is not applicable/relevant for your study

Specifically, in Pigg Party (Figure 1)—a platform in which each user is provided with a customizable avatar and a private room[26]—users in the experimental group were prompted to engage in the behavior of ringing a doorbell in another user's private room (Figure 2).

5-iii) Revisions and updating

Revisions and updating. Clearly mention the date and/or version number of the application/intervention (and comparator, if applicable) evaluated, or describe whether the intervention underwent major changes during the evaluation process, or whether the development and/or content was “frozen” during the trial. Describe dynamic components such as news feeds or changing content which may have an impact on the replicability of the intervention (for unexpected events see item 3b).

	1	2	3	4	5	
subitem not at all important	◉	○	○	○	○	essential

選択を解除

Does your paper address subitem 5-iii?

Copy and paste relevant sections from the manuscript (include quotes in quotation marks "like this" to indicate direct quotes from your manuscript), or elaborate on this item by providing additional information not in the ms, or briefly explain why the item is not applicable/relevant for your study

Since that element is not present in this experiment, it is not entered in the method.

5-iv) Quality assurance methods

Provide information on quality assurance methods to ensure accuracy and quality of information provided [1], if applicable.

	1	2	3	4	5	
subitem not at all important	◉	○	○	○	○	essential

選択を解除

Does your paper address subitem 5-iv?

Copy and paste relevant sections from the manuscript (include quotes in quotation marks "like this" to indicate direct quotes from your manuscript), or elaborate on this item by providing additional information not in the ms, or briefly explain why the item is not applicable/relevant for your study

Since that element is not present in this experiment, it is not entered in the method.

5-v) Ensure replicability by publishing the source code, and/or providing screenshots/screen-capture video, and/or providing flowcharts of the algorithms used

Ensure replicability by publishing the source code, and/or providing screenshots/screen-capture video, and/or providing flowcharts of the algorithms used. Replicability (i.e., other researchers should in principle be able to replicate the study) is a hallmark of scientific reporting.

	1	2	3	4	5	
subitem not at all important	○	○	○	○	◉	essential

選択を解除

Does your paper address subitem 5-v?

Copy and paste relevant sections from the manuscript (include quotes in quotation marks "like this" to indicate direct quotes from your manuscript), or elaborate on this item by providing additional information not in the ms, or briefly explain why the item is not applicable/relevant for your study

Figure 1. Players’ behavior in the Pigg Party.

5-vi) Digital preservation

Digital preservation: Provide the URL of the application, but as the intervention is likely to change or disappear over the course of the years; also make sure the intervention is archived (Internet Archive, webcitation.org, and/or publishing the source code or screenshots/videos alongside the article). As pages behind login screens cannot be archived, consider creating demo pages which are accessible without login.

	1	2	3	4	5	
subitem not at all important	◉	○	○	○	○	essential

選択を解除

Does your paper address subitem 5-vi?

Copy and paste relevant sections from the manuscript (include quotes in quotation marks "like this" to indicate direct quotes from your manuscript), or elaborate on this item by providing additional information not in the ms, or briefly explain why the item is not applicable/relevant for your study

The game can be accessed from anywhere in the world at any time, so there is no need to display a URL to access the game.

5-vii) Access

Access: Describe how participants accessed the application, in what setting/context, if they had to pay (or were paid) or not, whether they had to be a member of specific group. If known, describe how participants obtained “access to the platform and Internet” [1]. To ensure access for editors/reviewers/readers, consider to provide a “backdoor” login account or demo mode for reviewers/readers to explore the application (also important for archiving purposes, see vi).

	1	2	3	4	5	
subitem not at all important	◉	○	○	○	○	essential

選択を解除

Does your paper address subitem 5-vii? *

Copy and paste relevant sections from the manuscript (include quotes in quotation marks "like this" to indicate direct quotes from your manuscript), or elaborate on this item by providing additional information not in the ms, or briefly explain why the item is not applicable/relevant for your study

The game can be accessed from anywhere in the world at any time, so there is no need to display any login account to access the game.

5-viii) Mode of delivery, features/functionalities/components of the intervention and comparator, and the theoretical framework

Describe mode of delivery, features/functionalities/components of the intervention and comparator, and the theoretical framework [6] used to design them (instructional strategy [1], behaviour change techniques, persuasive features, etc., see e.g., [7, 8] for terminology). This includes an in-depth description of the content (including where it is coming from and who developed it) [1]," whether [and how] it is tailored to individual circumstances and allows users to track their progress and receive feedback" [6]. This also includes a description of communication delivery channels and – if computer-mediated communication is a component – whether communication was synchronous or asynchronous [6]. It also includes information on presentation strategies [1], including page design principles, average amount of text on pages, presence of hyperlinks to other resources, etc. [1].

	1	2	3	4	5	
subitem not at all important	○	○	○	○	◉	essential

選択を解除

Does your paper address subitem 5-viii? *

Copy and paste relevant sections from the manuscript (include quotes in quotation marks "like this" to indicate direct quotes from your manuscript), or elaborate on this item by providing additional information not in the ms, or briefly explain why the item is not applicable/relevant for your study

Online games improve the depressive and anxiety symptoms of game users[3]. Exposure therapy for anxiety symptoms is our second theoretical framework[16]. Individuals with social anxiety symptoms tend to become anxious because they have learned to perceive social situations as dangerous stimuli[17]. By repeatedly experiencing social situations with little danger, they learn to perceive such situations as safe stimuli, which in turn improves their anxiety symptoms[18]. Their re-learning of social situations has been confirmed in neuropsychological experiments[19], and the effectiveness of exposure therapy in improving social anxiety symptoms has been widely demonstrated[20]. Virtual reality exposure therapy, which constructs a virtual social community within online games and enables users to repeatedly experience social situations in the virtual community, has shown high intervention efficacy for social anxiety[21]. Virtual reality exposure therapy has been demonstrated to be effective for social anxiety symptoms in children[22], adolescents[23], and adults[24]; therefore, various virtual communities have been developed[25]. These findings suggest that repetitive experience of social situations within MMOGs improves the social anxiety of game users[6].

5-ix) Describe use parameters

Describe use parameters (e.g., intended “doses” and optimal timing for use). Clarify what instructions or recommendations were given to the user, e.g., regarding timing, frequency, heaviness of use, if any, or was the intervention used ad libitum.

	1	2	3	4	5	
subitem not at all important	○	○	○	○	◉	essential

選択を解除

Does your paper address subitem 5-ix?

Copy and paste relevant sections from the manuscript (include quotes in quotation marks "like this" to indicate direct quotes from your manuscript), or elaborate on this item by providing additional information not in the ms, or briefly explain why the item is not applicable/relevant for your study

Figure 4. Comparison of the number of times a friend's house bell was rung in a virtual space between the experimental and waitlist groups (LOG2).

5-x) Clarify the level of human involvement

Clarify the level of human involvement (care providers or health professionals, also technical assistance) in the e-intervention or as co-intervention (detail number and expertise of professionals involved, if any, as well as "type of assistance offered, the timing and frequency of the support, how it is initiated, and the medium by which the assistance is delivered". It may be necessary to distinguish between the level of human involvement required for the trial, and the level of human involvement required for a routine application outside of a RCT setting (discuss under item 21 – generalizability).

	1	2	3	4	5	
subitem not at all important	◉	○	○	○	○	essential

選択を解除

Does your paper address subitem 5-x?

Copy and paste relevant sections from the manuscript (include quotes in quotation marks "like this" to indicate direct quotes from your manuscript), or elaborate on this item by providing additional information not in the ms, or briefly explain why the item is not applicable/relevant for your study

Since that element is not present in this experiment, it is not entered in the method.

5-xi) Report any prompts/reminders used

Report any prompts/reminders used: Clarify if there were prompts (letters, emails, phone calls, SMS) to use the application, what triggered them, frequency etc. It may be necessary to distinguish between the level of prompts/reminders required for the trial, and the level of prompts/reminders for a routine application outside of a RCT setting (discuss under item 21 – generalizability).

	1	2	3	4	5	
subitem not at all important	○	○	○	○	●	essential

選択を解除

Does your paper address subitem 5-xi? *

Copy and paste relevant sections from the manuscript (include quotes in quotation marks "like this" to indicate direct quotes from your manuscript), or elaborate on this item by providing additional information not in the ms, or briefly explain why the item is not applicable/relevant for your study

The experimental group was prompted to visit another user’s room once per day and ring the bell on weekdays (Monday through Friday, excluding public holidays) (refer to Figure 2 for the method of ringing the bell) during the three-month experimental period (from December 16, 2023 to March 15, 2024). Additionally, on weekends (Saturday and Sunday) or public holidays, they were prompted to customize their avatar once per day (Multimedia Appendix 1 for the method of customization). Participants who accomplished these tasks on at least 18 days (60%) out of 30 days were awarded an additional bonus in the form of an Amazon or Google Play gift voucher worth 500 Japanese yen. As the experimental period spanned three months, gift vouchers worth a maximum of 1,500 Japanese yen were provided as additional bonuses per user.

5-xii) Describe any co-interventions (incl. training/support)

Describe any co-interventions (incl. training/support): Clearly state any interventions that are provided in addition to the targeted eHealth intervention, as ehealth intervention may not be designed as stand-alone intervention. This includes training sessions and support [1]. It may be necessary to distinguish between the level of training required for the trial, and the level of training for a routine application outside of a RCT setting (discuss under item 21 – generalizability.

	1	2	3	4	5	
subitem not at all important	◉	○	○	○	○	essential

選択を解除

Does your paper address subitem 5-xii? *

Copy and paste relevant sections from the manuscript (include quotes in quotation marks "like this" to indicate direct quotes from your manuscript), or elaborate on this item by providing additional information not in the ms, or briefly explain why the item is not applicable/relevant for your study

Since that element is not present in this experiment, it is not entered in the method.

6a) Completely defined pre-specified primary and secondary outcome measures, including how and when they were assessed

Does your paper address CONSORT subitem 6a? *

Copy and paste relevant sections from the manuscript (include quotes in quotation marks "like this" to indicate direct quotes from your manuscript), or elaborate on this item by providing additional information not in the ms, or briefly explain why the item is not applicable/relevant for your study

To assess depressive symptoms, the Quick Inventory of Depressive Symptomatology in Japanese version (QIDS-J) was employed[32,33]. The QIDS-J is a 16-item questionnaire that uses a 4-point Likert scale, with higher scores indicating greater severity of depression. The Cronbach's alpha coefficient for the QIDS-J at baseline was .86.

To assess social anxiety symptoms, the Brief Liebowitz Social Anxiety Scale in Japanese version (Brief LSAS-J) was used[6,34]. The Brief LSAS-J can measure social anxiety symptoms in both virtual and physical communities. It consists of a 14-item questionnaire for each community that assesses anxiety symptoms and avoidance behaviors. Higher scores on the Brief LSAS-J indicate greater severity of social anxiety symptoms in both virtual and physical communities. The Cronbach's alpha coefficients for the Brief LSAS-J at baseline in virtual and physical communities were .95 and .97, respectively.

6a-i) Online questionnaires: describe if they were validated for online use and apply CHERRIES items to describe how the questionnaires were designed/deployed

If outcomes were obtained through online questionnaires, describe if they were validated for online use and apply CHERRIES items to describe how the questionnaires were designed/deployed [9].

	1	2	3	4	5	
subitem not at all important	○	○	○	○	◉	essential

選択を解除

Does your paper address subitem 6a-i?

Copy and paste relevant sections from manuscript text

To assess depressive symptoms, the Quick Inventory of Depressive Symptomatology in Japanese version (QIDS-J) was employed[32,33]. The QIDS-J is a 16-item questionnaire that uses a 4-point Likert scale, with higher scores indicating greater severity of depression. The Cronbach's alpha coefficient for the QIDS-J at baseline was .86.

To assess social anxiety symptoms, the Brief Liebowitz Social Anxiety Scale in Japanese version (Brief LSAS-J) was used[6,34]. The Brief LSAS-J can measure social anxiety symptoms in both virtual and physical communities. It consists of a 14-item questionnaire for each community that assesses anxiety symptoms and avoidance behaviors. Higher scores on the Brief LSAS-J indicate greater severity of social anxiety symptoms in both virtual and physical communities. The Cronbach's alpha coefficients for the Brief LSAS-J at baseline in virtual and physical communities were .95 and .97, respectively.

6a-ii) Describe whether and how "use" (including intensity of use/dosage) was defined/measured/monitored

Describe whether and how "use" (including intensity of use/dosage) was defined/measured/monitored (logins, logfile analysis, etc.). Use/adoption metrics are important process outcomes that should be reported in any ehealth trial.

	1	2	3	4	5	
subitem not at all important	○	○	○	○	◉	essential

選択を解除

Does your paper address subitem 6a-ii?

Copy and paste relevant sections from manuscript text

To verify the frequency with which users rang the bell, it was measured monthly. Since the frequency of bell ringing is proportional to the number of friends and follows a power-law distribution, the value was calculated by adding one and then applying a base 2 logarithmic transformation.

Similarly, to verify the frequency with which users changed their avatar’s clothing, it was measured monthly. The resulting frequencies were then transformed by adding one and applying a base 2 logarithmic transformation.

6a-iii) Describe whether, how, and when qualitative feedback from participants was obtained

Describe whether, how, and when qualitative feedback from participants was obtained (e.g., through emails, feedback forms, interviews, focus groups).

	1	2	3	4	5	
subitem not at all important	○	○	○	○	◉	essential

選択を解除

Does your paper address subitem 6a-iii?

Copy and paste relevant sections from manuscript text

Participants completed questionnaires on demographic characteristics and outcome measures of mental health symptoms in the first wave (baseline, December 5–15, 2023). In the second wave (January 16–26, 2024), third wave (February 16–26, 2025), and fourth wave (March 16–26, 2025), they completed questionnaires solely on outcome measures of mental health symptoms. Participants who completed the questionnaires for the first and fourth waves within the designated period were provided with an Amazon gift voucher worth 500 Japanese yen (approximately 3 euros) or a Google Play gift voucher, whereas those who completed the questionnaires for the second and third waves within the designated period were provided with an Amazon gift voucher worth 200 Japanese yen (approximately 1 euro) or a Google Play gift voucher.

The experimental group was prompted to visit another user's room once per day and ring the bell on weekdays (Monday through Friday, excluding public holidays) (refer to Figure 2 for the method of ringing the bell) during the three-month experimental period (from December 16, 2023 to March 15, 2024). Additionally, on weekends (Saturday and Sunday) or public holidays, they were prompted to customize their avatar once per day (Multimedia Appendix 1 for the method of customization). Participants who accomplished these tasks on at least 18 days (60%) out of 30 days were awarded an additional bonus in the form of an Amazon or Google Play gift voucher worth 500 Japanese yen. As the experimental period spanned three months, gift vouchers worth a maximum of 1,500 Japanese yen were provided as additional bonuses per user.

In contrast, the waitlist group received no prompts during the experimental period. Within the waitlist group, 73% of the users were randomly selected each month and were awarded an additional bonus of either Amazon or Google Play gift vouchers worth 500 Japanese yen (Multimedia Appendix 2). Since the experimental period lasted for three months, gift vouchers worth a maximum of 1,500 Japanese yen were provided as additional bonuses per user.

6b) Any changes to trial outcomes after the trial commenced, with reasons

Does your paper address CONSORT subitem 6b? *

Copy and paste relevant sections from the manuscript (include quotes in quotation marks "like this" to indicate direct quotes from your manuscript), or elaborate on this item by providing additional information not in the ms, or briefly explain why the item is not applicable/relevant for your study

Since that element is not present in this experiment, it is not entered in the method.

7a) How sample size was determined

NPT: When applicable, details of whether and how the clustering by care provides or centers was addressed

7a-i) Describe whether and how expected attrition was taken into account when calculating the sample size

Describe whether and how expected attrition was taken into account when calculating the sample size.

	1	2	3	4	5	
subitem not at all important	○	○	○	○	◉	essential

選択を解除

Does your paper address subitem 7a-i?

Copy and paste relevant sections from manuscript title (include quotes in quotation marks "like this" to indicate direct quotes from your manuscript), or elaborate on this item by providing additional information not in the ms, or briefly explain why the item is not applicable/relevant for your study

The final number of participants required was 1,043 (based on an estimate for the latent growth model with d = 0.10, α = 0.05, β = 0.90)[35,36]. After recruiting 1,105 participants, it was deemed that a sufficient sample size had been reached, and further recruitment was terminated.

7b) When applicable, explanation of any interim analyses and stopping guidelines

Does your paper address CONSORT subitem 7b? *

Copy and paste relevant sections from the manuscript (include quotes in quotation marks "like this" to indicate direct quotes from your manuscript), or elaborate on this item by providing additional information not in the ms, or briefly explain why the item is not applicable/relevant for your study

Since that element is not present in this experiment, it is not entered in the method.

8a) Method used to generate the random allocation sequence
NPT: When applicable, how care providers were allocated to each trial group

Does your paper address CONSORT subitem 8a? *

Copy and paste relevant sections from the manuscript (include quotes in quotation marks "like this" to indicate direct quotes from your manuscript), or elaborate on this item by providing additional information not in the ms, or briefly explain why the item is not applicable/relevant for your study

Randomization and Masking
A total of 1,105 participants were randomly assigned to the experimental and waitlist groups in a 1:1 ratio (Figure 3). The Mersenne Twister method[31], which is implemented by default in R, was used for the random assignment. As the participants' Pigg Party tasks differed depending on the assigned group, assigned group masking was not implemented for the participants.

8b) Type of randomisation; details of any restriction (such as blocking and block size)

Does your paper address CONSORT subitem 8b? *

Copy and paste relevant sections from the manuscript (include quotes in quotation marks "like this" to indicate direct quotes from your manuscript), or elaborate on this item by providing additional information not in the ms, or briefly explain why the item is not applicable/relevant for your study

Since that element is not present in this experiment, it is not entered in the method.

9) Mechanism used to implement the random allocation sequence (such as sequentially numbered containers), describing any steps taken to conceal the sequence until interventions were assigned

Does your paper address CONSORT subitem 9? *

Copy and paste relevant sections from the manuscript (include quotes in quotation marks "like this" to indicate direct quotes from your manuscript), or elaborate on this item by providing additional information not in the ms, or briefly explain why the item is not applicable/relevant for your study

Randomization and Masking
A total of 1,105 participants were randomly assigned to the experimental and waitlist groups in a 1:1 ratio (Figure 3). The Mersenne Twister method[31], which is implemented by default in R, was used for the random assignment. As the participants' Pigg Party tasks differed depending on the assigned group, assigned group masking was not implemented for the participants.

10) Who generated the random allocation sequence, who enrolled participants, and who assigned participants to interventions

Does your paper address CONSORT subitem 10? *

Copy and paste relevant sections from the manuscript (include quotes in quotation marks "like this" to indicate direct quotes from your manuscript), or elaborate on this item by providing additional information not in the ms, or briefly explain why the item is not applicable/relevant for your study

Randomization and Masking
A total of 1,105 participants were randomly assigned to the experimental and waitlist groups in a 1:1 ratio (Figure 3). The Mersenne Twister method[31], which is implemented by default in R, was used for the random assignment. As the participants' Pigg Party tasks differed depending on the assigned group, assigned group masking was not implemented for the participants.

11a) If done, who was blinded after assignment to interventions (for example, participants, care providers, those assessing outcomes) and how

NPT: Whether or not administering co-interventions were blinded to group assignment

11a-i) Specify who was blinded, and who wasn't

Specify who was blinded, and who wasn't. Usually, in web-based trials it is not possible to blind the participants [1, 3] (this should be clearly acknowledged), but it may be possible to blind outcome assessors, those doing data analysis or those administering co-interventions (if any).

	1	2	3	4	5	
subitem not at all important	○	○	○	○	◉	essential

選択を解除

Does your paper address subitem 11a-i? *

Copy and paste relevant sections from the manuscript (include quotes in quotation marks "like this" to indicate direct quotes from your manuscript), or elaborate on this item by providing additional information not in the ms, or briefly explain why the item is not applicable/relevant for your study

Randomization and Masking
A total of 1,105 participants were randomly assigned to the experimental and waitlist groups in a 1:1 ratio (Figure 3). The Mersenne Twister method[31], which is implemented by default in R, was used for the random assignment. As the participants' Pigg Party tasks differed depending on the assigned group, assigned group masking was not implemented for the participants.

11a-ii) Discuss e.g., whether participants knew which intervention was the "intervention of interest" and which one was the "comparator"

Informed consent procedures (4a-ii) can create biases and certain expectations - discuss e.g., whether participants knew which intervention was the "intervention of interest" and which one was the "comparator".

	1	2	3	4	5	
subitem not at all important	○	○	○	○	◉	essential

選択を解除

Does your paper address subitem 11a-ii?

Copy and paste relevant sections from the manuscript (include quotes in quotation marks "like this" to indicate direct quotes from your manuscript), or elaborate on this item by providing additional information not in the ms, or briefly explain why the item is not applicable/relevant for your study

Randomization and Masking
A total of 1,105 participants were randomly assigned to the experimental and waitlist groups in a 1:1 ratio (Figure 3). The Mersenne Twister method[31], which is implemented by default in R, was used for the random assignment. As the participants' Pigg Party tasks differed depending on the assigned group, assigned group masking was not implemented for the participants.

11b) If relevant, description of the similarity of interventions

(this item is usually not relevant for ehealth trials as it refers to similarity of a placebo or sham intervention to a active medication/intervention)

Does your paper address CONSORT subitem 11b? *

Copy and paste relevant sections from the manuscript (include quotes in quotation marks "like this" to indicate direct quotes from your manuscript), or elaborate on this item by providing additional information not in the ms, or briefly explain why the item is not applicable/relevant for your study

Since that element is not present in this experiment, it is not entered in the method.

12a) Statistical methods used to compare groups for primary and secondary outcomes

NPT: When applicable, details of whether and how the clustering by care providers or centers was addressed

Does your paper address CONSORT subitem 12a? *

Copy and paste relevant sections from the manuscript (include quotes in quotation marks "like this" to indicate direct quotes from your manuscript), or elaborate on this item by providing additional information not in the ms, or briefly explain why the item is not applicable/relevant for your study

The final number of participants required was 1,043 (based on an estimate for the latent growth model with d = 0.10, α = 0.05, β = 0.90)[35,36]. After recruiting 1,105 participants, it was deemed that a sufficient sample size had been reached, and further recruitment was terminated.

Latent Growth Curve Modeling (LGCM) was used in this study. The current structural equations of the LGCM accounted for the effect of gender, following a previous study[37]. The equations are as follows:
Y[it]=B0[i]+B1[i]F[t]+E2[it]
B0[i]=B00+B0exp*Gexp[i]+B0female*Gfemale[i]+E0[i]
B1[i]=B10+B1exp*Gexp[i]+B1female*Gfemale[i]+E1[i]

Where Y[it] represents the outcome measures (depressive symptoms, social anxiety symptoms) at time t for participant i. B0[i] and B1[i] are the intercept and slope contributing to Y[it] for participant i. E2[it] is the error contributing to Y[it] at time t for participant i. In line with previous studies[38,39], F[t] is obtained by logarithmically transforming the time of 4t weeks after adding 1. Specifically, for 4t = 0, 4, 8, and 12 weeks, F[t] corresponds to 0.000, 1.000, 1.585, and 2.000, respectively.

The intercept B0[i] is influenced by B00, B0exp, B0female, and E0, which represent the intercept for the intercept, intercept effect of the experimental group, intercept effect of the female group, and intercept effects of individuals, respectively. Gexp[i] is a group variable indicating whether participant i belongs to the experimental group (1 if yes, 0 if no). Similarly, Gfemale[i] is another group variable indicating the sex of the participant (1 for females, 0 for no females).

The slope B1[i] is influenced by B10, B1exp, B1female, and E1, which represent the intercept for the slope, slope effect of the experimental group, slope effect of the female group, and slope effect of individuals, respectively. B1exp represents the time-variant effects of the experimental group on the outcome measure Y[it]. Hence, this effect represents the intervention effects of the experimental group on the outcome measures. The intervention effects were analyzed using 95% confidence intervals and Wald tests. The Widely Applicable Bayesian Information Criterion (WAIC) and Leave-One-Out cross validation (LOO) were used as indicators of the model's overall fit.

12a-i) Imputation techniques to deal with attrition / missing values

Imputation techniques to deal with attrition / missing values: Not all participants will use the intervention/comparator as intended and attrition is typically high in ehealth trials. Specify how participants who did not use the application or dropped out from the trial were treated in the statistical analysis (a complete case analysis is strongly discouraged, and simple imputation techniques such as LOCF may also be problematic [4]).

	1	2	3	4	5	
subitem not at all important	○	○	○	○	◉	essential

選択を解除

Does your paper address subitem 12a-i? *

Copy and paste relevant sections from the manuscript (include quotes in quotation marks "like this" to indicate direct quotes from your manuscript), or elaborate on this item by providing additional information not in the ms, or briefly explain why the item is not applicable/relevant for your study

In accordance with clinical trial guidelines[40], intention-to-treat analyses were performed. Full Information Maximum Likelihood (FIML) was used to handle missing data[41], as it is commonly used for estimating missing data in clinical trials[37]. During the FIML procedure, 0.0001 was added to the diagonal matrix to ensure that the covariance matrix remained positive. In addition, instead of an inverse matrix, a pseudo-inverse matrix was used to manage errors when the inverse matrix did not exist. The initial values were set as the mean of each variable, and the optimization process was accelerated using the Limited-memory Broyden-Fletcher-Goldfarb-Shanno algorithm-bound optimization approach[42].

12b) Methods for additional analyses, such as subgroup analyses and adjusted analyses

Does your paper address CONSORT subitem 12b? *

Copy and paste relevant sections from the manuscript (include quotes in quotation marks "like this" to indicate direct quotes from your manuscript), or elaborate on this item by providing additional information not in the ms, or briefly explain why the item is not applicable/relevant for your study

Since that element is not present in this experiment, it is not entered into the method.

X26) REB/IRB Approval and Ethical Considerations [recommended as subheading under "Methods"] (not a CONSORT item)

X26-i) Comment on ethics committee approval

	1	2	3	4	5	
subitem not at all important	○	○	○	○	◉	essential

選択を解除

Does your paper address subitem X26-i?

Copy and paste relevant sections from the manuscript (include quotes in quotation marks "like this" to indicate direct quotes from your manuscript), or elaborate on this item by providing additional information not in the ms, or briefly explain why the item is not applicable/relevant for your study

Ethical Considerations
This study was approved by the Ethics Committee of the National University A in Japan as of December 1, 2023 (registration number 301). Informed consent was obtained from all the participants in this study. All procedures were conducted in accordance with the revised 1964 Helsinki Declaration and its later amendments, or comparable ethical standards.

x26-ii) Outline informed consent procedures

Outline informed consent procedures e.g., if consent was obtained offline or online (how? Checkbox, etc.?), and what information was provided (see 4a-ii). See [6] for some items to be included in informed consent documents.

	1	2	3	4	5	
subitem not at all important	○	○	○	○	◉	essential

選択を解除

Does your paper address subitem X26-ii?

Copy and paste relevant sections from the manuscript (include quotes in quotation marks "like this" to indicate direct quotes from your manuscript), or elaborate on this item by providing additional information not in the ms, or briefly explain why the item is not applicable/relevant for your study

Participants
The participants of this study were users who had been using the Pigg Party before September 15, 2023. On December 5, 2023, the administrator of the Pigg Party emailed the users requesting their participation in the study. By December 15, 2023, 1,331 users had expressed their willingness to participate, and then their eligibility for participation was assessed. As a result, 226 users were removed from the participants; among these, 175 users had withdrawn their consent to participate in the experiment, and after the recruitment period, 51 users requested to participate. The final participants consisted of 1,105 Pigg Party users.

X26-iii) Safety and security procedures

Safety and security procedures, incl. privacy considerations, and any steps taken to reduce the likelihood or detection of harm (e.g., education and training, availability of a hotline)

	1	2	3	4	5	
subitem not at all important	◉	○	○	○	○	essential

選択を解除

Does your paper address subitem X26-iii?

Copy and paste relevant sections from the manuscript (include quotes in quotation marks "like this" to indicate direct quotes from your manuscript), or elaborate on this item by providing additional information not in the ms, or briefly explain why the item is not applicable/relevant for your study

Since that element is not present in this experiment, it is not entered into the method.

RESULTS

13a) For each group, the numbers of participants who were randomly assigned, received intended treatment, and were analysed for the primary outcome

NPT: The number of care providers or centers performing the intervention in each group and the number of patients treated by each care provider in each center

Does your paper address CONSORT subitem 13a? *

Copy and paste relevant sections from the manuscript (include quotes in quotation marks "like this" to indicate direct quotes from your manuscript), or elaborate on this item by providing additional information not in the ms, or briefly explain why the item is not applicable/relevant for your study

We compared mental health symptoms between the experimental and waitlist groups. The experimental group exhibited a significantly greater improvement in depressive symptoms compared to the waitlist group (Table 2). Figure 5 further illustrates that the experimental group demonstrated a consistent improvement in depressive symptoms. These findings support Hypothesis 1. In contrast, there was no significant difference between the experimental and waitlist groups regarding social anxiety in both virtual and physical communities (Table 2). Thus, these results did not support Hypothesis 2.

13b) For each group, losses and exclusions after randomisation, together with reasons

Does your paper address CONSORT subitem 13b? (NOTE: Preferably, this is shown in a CONSORT flow diagram) *

Copy and paste relevant sections from the manuscript (include quotes in quotation marks "like this" to indicate direct quotes from your manuscript), or elaborate on this item by providing additional information not in the ms, or briefly explain why the item is not applicable/relevant for your study

In accordance with clinical trial guidelines[40], intention-to-treat analyses were performed. Full Information Maximum Likelihood (FIML) was used to handle missing data[41], as it is commonly used for estimating missing data in clinical trials[37]. During the FIML procedure, 0.0001 was added to the diagonal matrix to ensure that the covariance matrix remained positive. In addition, instead of an inverse matrix, a pseudo-inverse matrix was used to manage errors when the inverse matrix did not exist. The initial values were set as the mean of each variable, and the optimization process was accelerated using the Limited-memory Broyden-Fletcher-Goldfarb-Shanno algorithm-bound optimization approach[42].

13b-i) Attrition diagram

Strongly recommended: An attrition diagram (e.g., proportion of participants still logging in or using the intervention/comparator in each group plotted over time, similar to a survival curve) or other figures or tables demonstrating usage/dose/engagement.

	1	2	3	4	5	
subitem not at all important	○	○	○	○	◉	essential

選択を解除

Does your paper address subitem 13b-i?

Copy and paste relevant sections from the manuscript or cite the figure number if applicable (include quotes in quotation marks "like this" to indicate direct quotes from your manuscript), or elaborate on this item by providing additional information not in the ms, or briefly explain why the item is not applicable/relevant for your study

Figure 3. Participant flow diagram.

14a) Dates defining the periods of recruitment and follow-up

Does your paper address CONSORT subitem 14a? *

Copy and paste relevant sections from the manuscript (include quotes in quotation marks "like this" to indicate direct quotes from your manuscript), or elaborate on this item by providing additional information not in the ms, or briefly explain why the item is not applicable/relevant for your study

Participants
The participants of this study were users who had been using the Pigg Party before September 15, 2023. On December 5, 2023, the administrator of the Pigg Party emailed the users requesting their participation in the study. By December 15, 2023, 1,331 users had expressed their willingness to participate, and then their eligibility for participation was assessed. As a result, 226 users were removed from the participants; among these, 175 users had withdrawn their consent to participate in the experiment, and after the recruitment period, 51 users requested to participate. The final participants consisted of 1,105 Pigg Party users.

14a-i) Indicate if critical “secular events” fell into the study period

Indicate if critical “secular events” fell into the study period, e.g., significant changes in Internet resources available or “changes in computer hardware or Internet delivery resources”

	1	2	3	4	5	
subitem not at all important	◉	○	○	○	○	essential

選択を解除

Does your paper address subitem 14a-i?

Copy and paste relevant sections from the manuscript (include quotes in quotation marks "like this" to indicate direct quotes from your manuscript), or elaborate on this item by providing additional information not in the ms, or briefly explain why the item is not applicable/relevant for your study

Since that element is not present in this experiment, it is not entered into the method.

14b) Why the trial ended or was stopped (early)

Does your paper address CONSORT subitem 14b? *

Copy and paste relevant sections from the manuscript (include quotes in quotation marks "like this" to indicate direct quotes from your manuscript), or elaborate on this item by providing additional information not in the ms, or briefly explain why the item is not applicable/relevant for your study

Since that element is not present in this experiment, it is not entered into the method.

15) A table showing baseline demographic and clinical characteristics for each group

NPT: When applicable, a description of care providers (case volume, qualification, expertise, etc.) and centers (volume) in each group

Does your paper address CONSORT subitem 15? *

Copy and paste relevant sections from the manuscript (include quotes in quotation marks "like this" to indicate direct quotes from your manuscript), or elaborate on this item by providing additional information not in the ms, or briefly explain why the item is not applicable/relevant for your study

Table 1. Comparison of demographic variables between the experimental and waitlist groups. a-c

15-i) Report demographics associated with digital divide issues

In ehealth trials it is particularly important to report demographics associated with digital divide issues, such as age, education, gender, social-economic status, computer/Internet/ehealth literacy of the participants, if known.

	1	2	3	4	5	
subitem not at all important	○	○	○	○	◉	essential

選択を解除

Does your paper address subitem 15-i? *

Copy and paste relevant sections from the manuscript (include quotes in quotation marks "like this" to indicate direct quotes from your manuscript), or elaborate on this item by providing additional information not in the ms, or briefly explain why the item is not applicable/relevant for your study

Table 1. Comparison of demographic variables between the experimental and waitlist groups. a-c

16) For each group, number of participants (denominator) included in each analysis and whether the analysis was by original assigned groups

16-i) Report multiple “denominators” and provide definitions

Report multiple “denominators” and provide definitions: Report N’s (and effect sizes) “across a range of study participation [and use] thresholds” [1], e.g., N exposed, N consented, N used more than x times, N used more than y weeks, N participants “used” the intervention/comparator at specific pre-defined time points of interest (in absolute and relative numbers per group). Always clearly define “use” of the intervention.

	1	2	3	4	5	
subitem not at all important	○	○	○	○	◉	essential

選択を解除

Does your paper address subitem 16-i? *

Copy and paste relevant sections from the manuscript (include quotes in quotation marks "like this" to indicate direct quotes from your manuscript), or elaborate on this item by providing additional information not in the ms, or briefly explain why the item is not applicable/relevant for your study

In accordance with clinical trial guidelines[40], intention-to-treat analyses were performed.

16-ii) Primary analysis should be intent-to-treat

Primary analysis should be intent-to-treat, secondary analyses could include comparing only “users”, with the appropriate caveats that this is no longer a randomized sample (see 18-i).

	1	2	3	4	5	
subitem not at all important	○	○	○	○	◉	essential

選択を解除

Does your paper address subitem 16-ii?

Copy and paste relevant sections from the manuscript (include quotes in quotation marks "like this" to indicate direct quotes from your manuscript), or elaborate on this item by providing additional information not in the ms, or briefly explain why the item is not applicable/relevant for your study

In accordance with clinical trial guidelines[40], intention-to-treat analyses were performed.

17a) For each primary and secondary outcome, results for each group, and the estimated effect size and its precision (such as 95% confidence interval)

Does your paper address CONSORT subitem 17a? *

Copy and paste relevant sections from the manuscript (include quotes in quotation marks "like this" to indicate direct quotes from your manuscript), or elaborate on this item by providing additional information not in the ms, or briefly explain why the item is not applicable/relevant for your study

Table 2. Intention-to-treat analysis of latent growth curve modeling of depressive and social anxiety symptoms. a-e

17a-i) Presentation of process outcomes such as metrics of use and intensity of use

In addition to primary/secondary (clinical) outcomes, the presentation of process outcomes such as metrics of use and intensity of use (dose, exposure) and their operational definitions is critical. This does not only refer to metrics of attrition (13-b) (often a binary variable), but also to more continuous exposure metrics such as “average session length”. These must be accompanied by a technical description how a metric like a “session” is defined (e.g., timeout after idle time) [1] (report under item 6a).

	1	2	3	4	5	
subitem not at all important	○	○	○	○	◉	essential

選択を解除

Does your paper address subitem 17a-i?

Copy and paste relevant sections from the manuscript (include quotes in quotation marks "like this" to indicate direct quotes from your manuscript), or elaborate on this item by providing additional information not in the ms, or briefly explain why the item is not applicable/relevant for your study

In addition, we confirmed that the manipulation applied to the experimental group was conducted appropriately. The experimental group exhibited a significantly higher number of bell-ringing behaviors than the waitlist group (Table 2)

17b) For binary outcomes, presentation of both absolute and relative effect sizes is recommended

Does your paper address CONSORT subitem 17b? *

Copy and paste relevant sections from the manuscript (include quotes in quotation marks "like this" to indicate direct quotes from your manuscript), or elaborate on this item by providing additional information not in the ms, or briefly explain why the item is not applicable/relevant for your study

Since that element is not present in this experiment, it is not entered into the method.

18) Results of any other analyses performed, including subgroup analyses and adjusted analyses, distinguishing pre-specified from exploratory

Does your paper address CONSORT subitem 18? *

Copy and paste relevant sections from the manuscript (include quotes in quotation marks "like this" to indicate direct quotes from your manuscript), or elaborate on this item by providing additional information not in the ms, or briefly explain why the item is not applicable/relevant for your study

Since that element is not present in this experiment, it is not entered into the method.

18-i) Subgroup analysis of comparing only users

A subgroup analysis of comparing only users is not uncommon in ehealth trials, but if done, it must be stressed that this is a self-selected sample and no longer an unbiased sample from a randomized trial (see 16-iii).

	1	2	3	4	5	
subitem not at all important	◉	○	○	○	○	essential

選択を解除

Does your paper address subitem 18-i?

Copy and paste relevant sections from the manuscript (include quotes in quotation marks "like this" to indicate direct quotes from your manuscript), or elaborate on this item by providing additional information not in the ms, or briefly explain why the item is not applicable/relevant for your study

Since that element is not present in this experiment, it is not entered into the method.

19) All important harms or unintended effects in each group

(for specific guidance see CONSORT for harms)

Does your paper address CONSORT subitem 19? *

Copy and paste relevant sections from the manuscript (include quotes in quotation marks "like this" to indicate direct quotes from your manuscript), or elaborate on this item by providing additional information not in the ms, or briefly explain why the item is not applicable/relevant for your study

Since that element is not present in this experiment, it is not entered into the method.

19-i) Include privacy breaches, technical problems

Include privacy breaches, technical problems. This does not only include physical “harm” to participants, but also incidents such as perceived or real privacy breaches [1], technical problems, and other unexpected/unintended incidents. “Unintended effects” also includes unintended positive effects [2].

	1	2	3	4	5	
subitem not at all important	◉	○	○	○	○	essential

選択を解除

Does your paper address subitem 19-i?

Copy and paste relevant sections from the manuscript (include quotes in quotation marks "like this" to indicate direct quotes from your manuscript), or elaborate on this item by providing additional information not in the ms, or briefly explain why the item is not applicable/relevant for your study

Since that element is not present in this experiment, it is not entered into the method.

19-ii) Include qualitative feedback from participants or observations from staff/researchers

Include qualitative feedback from participants or observations from staff/researchers, if available, on strengths and shortcomings of the application, especially if they point to unintended/unexpected effects or uses. This includes (if available) reasons for why people did or did not use the application as intended by the developers.

	1	2	3	4	5	
subitem not at all important	(●)	○	○	○	○	essential

選択を解除

Does your paper address subitem 19-ii?

Copy and paste relevant sections from the manuscript (include quotes in quotation marks "like this" to indicate direct quotes from your manuscript), or elaborate on this item by providing additional information not in the ms, or briefly explain why the item is not applicable/relevant for your study

Since that element is not present in this experiment, it is not entered into the method.

DISCUSSION

22) Interpretation consistent with results, balancing benefits and harms, and considering other relevant evidence

NPT: In addition, take into account the choice of the comparator, lack of or partial blinding, and unequal expertise of care providers or centers in each group

22-i) Restate study questions and summarize the answers suggested by the data, starting with primary outcomes and process outcomes (use)

Restate study questions and summarize the answers suggested by the data, starting with primary outcomes and process outcomes (use).

	1	2	3	4	5	
subitem not at all important	○	○	○	○	◉	essential

選択を解除

Does your paper address subitem 22-i? *

Copy and paste relevant sections from the manuscript (include quotes in quotation marks "like this" to indicate direct quotes from your manuscript), or elaborate on this item by providing additional information not in the ms, or briefly explain why the item is not applicable/relevant for your study

Principal Results
This study investigated whether mental health symptoms can be improved through interventions on MMOGs by conducting a clinical trial with over 1,000 participants. Consistent with previous findings[2,5], our results confirm that depressive symptoms can be alleviated through interventions on MMOGs. Given the large user base of MMOGs, such interventions may significantly contribute to reducing depressive symptoms on a broad scale[1]. Additionally, since the intervention was integrated as part of the in-game tasks, users could potentially experience an improvement in depressive symptoms while enjoying the game. Previous studies have used specific applications to improve depressive symptoms, which imposed a certain burden on users in terms of the intervention[12–15]. In contrast, since the participants in this study engaged with an MMOG interface they were already familiar with, the burden on users was minimal. This suggests that MMOG-based interventions could serve as an effective and accessible approach to alleviate depressive symptoms without imposing additional burden[43].

22-ii) Highlight unanswered new questions, suggest future research

Highlight unanswered new questions, suggest future research.

	1	2	3	4	5	
subitem not at all important	○	○	○	○	◉	essential

選択を解除

Does your paper address subitem 22-ii?

Copy and paste relevant sections from the manuscript (include quotes in quotation marks "like this" to indicate direct quotes from your manuscript), or elaborate on this item by providing additional information not in the ms, or briefly explain why the item is not applicable/relevant for your study

Second, the platform was limited to Pigg Party[26]. Given that immersion is a key factor in exposure therapy[25], further research is needed to determine whether the finding can be replicated in MMOGs with a virtual reality headset.

20) Trial limitations, addressing sources of potential bias, imprecision, and, if relevant, multiplicity of analyses

20-i) Typical limitations in ehealth trials

Typical limitations in ehealth trials: Participants in ehealth trials are rarely blinded. Ehealth trials often look at a multiplicity of outcomes, increasing risk for a Type I error. Discuss biases due to non-use of the intervention/usability issues, biases through informed consent procedures, unexpected events.

	1	2	3	4	5	
subitem not at all important	○	○	○	○	◉	essential

選択を解除

Does your paper address subitem 20-i? *

Copy and paste relevant sections from the manuscript (include quotes in quotation marks "like this" to indicate direct quotes from your manuscript), or elaborate on this item by providing additional information not in the ms, or briefly explain why the item is not applicable/relevant for your study

Limitations
This study had two main limitations. First, despite the random assignment of participants, the experimental group had a significantly lower proportion of females compared to the waitlist group. The gender imbalance may have influenced the results. Second, the platform was limited to Pigg Party[26]. Given that immersion is a key factor in exposure therapy[25], further research is needed to determine whether the finding can be replicated in MMOGs with a virtual reality headset.

21) Generalisability (external validity, applicability) of the trial findings

NPT: External validity of the trial findings according to the intervention, comparators, patients, and care providers or centers involved in the trial

21-i) Generalizability to other populations

Generalizability to other populations: In particular, discuss generalizability to a general Internet population, outside of a RCT setting, and general patient population, including applicability of the study results for other organizations

	1	2	3	4	5	
subitem not at all important	○	○	○	○	●	essential

選択を解除

Does your paper address subitem 21-i?

Copy and paste relevant sections from the manuscript (include quotes in quotation marks "like this" to indicate direct quotes from your manuscript), or elaborate on this item by providing additional information not in the ms, or briefly explain why the item is not applicable/relevant for your study

This study, with a randomized controlled trial with over 1,000 participants, highlights the potential of MMOG-based interventions to alleviate depressive symptoms. Notably, the MMOG used in this study was developed for leisure purposes rather than for therapeutic purposes[26]. This suggests that users experienced improvements in depressive symptoms while enjoying a leisure activity[4]. By extending this approach to clinical trials on other MMOGs, it may become possible to improve depressive symptoms in a wider population through the accessible interface of online games[3,43].

21-ii) Discuss if there were elements in the RCT that would be different in a routine application setting

Discuss if there were elements in the RCT that would be different in a routine application setting (e.g., prompts/reminders, more human involvement, training sessions or other co-interventions) and what impact the omission of these elements could have on use, adoption, or outcomes if the intervention is applied outside of a RCT setting.

	1	2	3	4	5	
subitem not at all important	◉	○	○	○	○	essential

選択を解除

Does your paper address subitem 21-ii?

Copy and paste relevant sections from the manuscript (include quotes in quotation marks "like this" to indicate direct quotes from your manuscript), or elaborate on this item by providing additional information not in the ms, or briefly explain why the item is not applicable/relevant for your study

Since that element is not present in this experiment, it is not entered into the method.

OTHER INFORMATION

23) Registration number and name of trial registry

Does your paper address CONSORT subitem 23? *

Copy and paste relevant sections from the manuscript (include quotes in quotation marks "like this" to indicate direct quotes from your manuscript), or elaborate on this item by providing additional information not in the ms, or briefly explain why the item is not applicable/relevant for your study

Trial Registration: WDB9U (DOI: 10.17605/OSF.IO/WDB9U)

24) Where the full trial protocol can be accessed, if available

Does your paper address CONSORT subitem 24? *

Cite a Multimedia Appendix, other reference, or copy and paste relevant sections from the manuscript (include quotes in quotation marks "like this" to indicate direct quotes from your manuscript), or elaborate on this item by providing additional information not in the ms, or briefly explain why the item is not applicable/relevant for your study

Trial Registration: WDB9U (DOI: 10.17605/OSF.IO/WDB9U)

25) Sources of funding and other support (such as supply of drugs), role of funders

Does your paper address CONSORT subitem 25? *

Copy and paste relevant sections from the manuscript (include quotes in quotation marks "like this" to indicate direct quotes from your manuscript), or elaborate on this item by providing additional information not in the ms, or briefly explain why the item is not applicable/relevant for your study

Funding statement
Kenji Yokotani was funded by a grant from CyberAgent, Inc. (akblab-0005).

X27) Conflicts of Interest (not a CONSORT item)

X27-i) State the relation of the study team towards the system being evaluated

In addition to the usual declaration of interests (financial or otherwise), also state the relation of the study team towards the system being evaluated, i.e., state if the authors/evaluators are distinct from or identical with the developers/sponsors of the intervention.

	1	2	3	4	5	
subitem not at all important	○	○	○	○	◉	essential

選択を解除

Does your paper address subitem X27-i?

Copy and paste relevant sections from the manuscript (include quotes in quotation marks "like this" to indicate direct quotes from your manuscript), or elaborate on this item by providing additional information not in the ms, or briefly explain why the item is not applicable/relevant for your study

Conflict of Interests
Author(s') disclosure (Conflict of Interest) statement(s)
Kenji Yokotani was funded by CyberAgent, Inc. Masanori Takano was an employee of CyberAgent, Inc.

About the CONSORT EHEALTH checklist

As a result of using this checklist, did you make changes in your manuscript? *

- ○ yes, major changes
- ○ yes, minor changes
- ◉ no

What were the most important changes you made as a result of using this checklist?

回答を入力

How much time did you spend on going through the checklist INCLUDING making changes in your manuscript *

It took from 30 minutes to an hour.

As a result of using this checklist, do you think your manuscript has improved? *

- ( ) yes
- (•) no
- ( ) その他:

Would you like to become involved in the CONSORT EHEALTH group?

This would involve for example becoming involved in participating in a workshop and writing an "Explanation and Elaboration" document

- ( ) yes
- (•) no
- ( ) その他:

選択を解除

Any other comments or questions on CONSORT EHEALTH

回答を入力

STOP - Save this form as PDF before you click submit

To generate a record that you filled in this form, we recommend to generate a PDF of this page (on a Mac, simply select "print" and then select "print as PDF") before you submit it.

When you submit your (revised) paper to JMIR, please upload the PDF as supplementary file.

Don't worry if some text in the textboxes is cut off, as we still have the complete information in our database. Thank you!

Final step: Click submit !

Click submit so we have your answers in our database!

送信 フォームをクリア

Google フォームでパスワードを送信しないでください。

このコンテンツは Google が作成または承認したものではありません。 - 利用規約 - プライバシー ポリシー

Does this form look suspicious? 報告

Google フォーム
